# Mimicking Real Catalysts: Model Stepped Nickel Surfaces in Furfural Catalysis—Insights into Adsorption, Reactivity, and Defect-Driven Conversion Pathways

**DOI:** 10.1021/acs.jpclett.5c00066

**Published:** 2025-03-17

**Authors:** Sotirios Tsatsos, Georgios Kyriakou

**Affiliations:** Department of Chemical Engineering, University of Patras, Caratheodory 1, Patras GR 26504, Greece

## Abstract

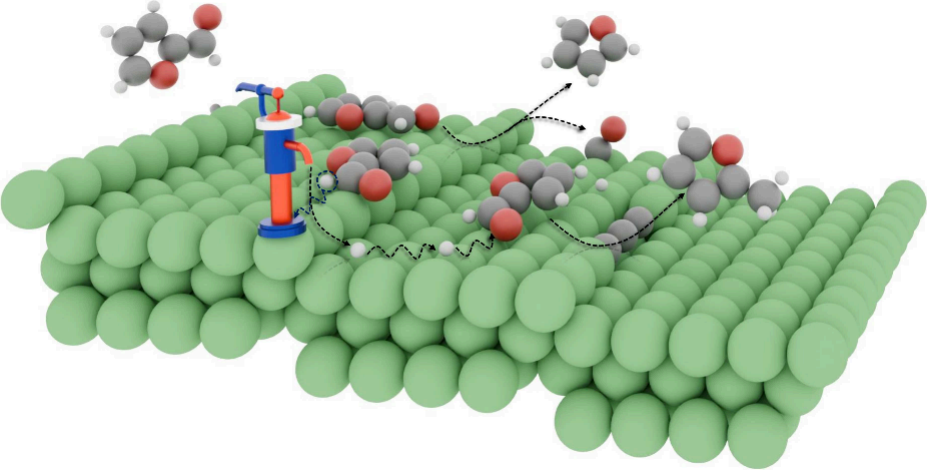

The catalytic conversion of furanic compounds into renewable chemicals is essential for sustainable manufacturing. Here, we report a unique self-hydrogenation pathway of furfural to 2-methylfuran on Ni(119) surface, showing how steps and nickel carbides govern reaction selectivity. Thermal desorption and spectroscopic measurements reveal that furfural undergoes decarbonylation to furan on terraces, while step sites act as “hydrogen transfer pumps”, abstracting hydrogen from furfural and facilitating its diffusion to terrace-bound molecules, thereby promoting selective hydrogenation to 2-methylfuran. Moreover, the surface-bound hydrogen enhances hydrogenolysis, with product selectivity closely connected to hydrogen concentration. DFT calculations show a preference for the top step edges, where strong bonding and electron redistribution stabilize intermediates and promote catalytic transformations. We further demonstrate how these insights provide a framework for designing advanced catalysts through surface structure optimization. By linking model catalysts with real-world applications, this approach enables the development of efficient and selective catalysts tailored for biomass conversion.

The conversion of biomass into fuels and valuable chemicals offers a promising approach to sustainable manufacturing and renewable energy supply.^1,2^ To a large extent, biomass upgrading relies on catalytic processes, involving the selective transformation of complex organic molecules with multiple oxygen functional groups into various products of interest.^3,4^ In this respect, optimizing catalyst structures and their electronic environment is essential for advancing modern biochemical industries and, in turn, enhancing sustainability. Furfural (FUR), a key biomass-derived molecule, is industrially significant as a precursor for valuable products like furan, furfuryl alcohol (FAL), tetrahydrofurfuryl alcohol (THFA), and 2-methylfuran (2MF).^5,6^ Catalysts such as Cu,^7,8^ Ni,^9−12^ Pd,^13−15^ Pt,^16−18^ and Ru^19^ offer distinct selectivities: Ni excels in hydrogenation but favors ring-opening, while Pd and Pt reduce ring interactions. The hydrogenation of FUR in industry occurs in both liquid and gas phases. In gas-phase hydrogenation, primarily used for FAL production, copper-based catalysts (Cu/SiO_2_, Cu/Cr_2_O_3_) are typically employed at 200–250 °C and 2–4 MPa H_2_. Conversely, Ni, Pd, and Ru are more common in gas-phase hydrodeoxygenation (HDO), converting FUR to 2MF via C–O bond cleavage.^5,20,21^ Liquid-phase hydrogenation, which is used to produce FAL, THFA, and tetrahydrofuran (THF), operates under 100–200 °C and 3–5 MPa H_2_ conditions with Ni, Pd, Ru, or Pt catalysts.^5,6,22^ On model systems such as Cu(111), Ni(111), and NiCu surfaces, FUR undergoes enhanced hydrodeoxygenation (HDO) due to its stronger interactions with the carbonyl group, which promote selective hydrogenation that retains aromaticity and limits decarbonylation.^23,24^

However, the performance of dispersed metal catalysts is greatly influenced by the size and structure of metal nanoparticles,^25^ as these nanoparticles offer a variety of active sites with different electrophilicities, coordination, and electronic properties, enhancing catalytic activity and selectivity.^26−28^ In the case of the catalytic hydrogenation of FUR, it has been reported that particle size and shape significantly impact the reaction mechanism and selectivity^29^ as shown for Pt,^30^ Ni^31,32^ and Ru^29^ metal nanoparticles. Clearly, the type of active sites present on the nanoparticle surface has a distinct effect on the adsorption and hydrogenation of FUR and its intermediates. To enhance catalytic performance, it is essential to understand selective adsorption and the preferences of different active sites for FUR and its intermediates, addressing an open problem in the literature.^33^ In this context, high-index surfaces can serve as effective models to isolate and study the behavior of individual active sites that are abundant on practical catalysts. Prior studies on acetylene adsorption on Ni(755)^34^ highlight the reactivity of step sites in C–H bond activation, while Luneau et al.^35^ emphasized their role in hydrogenation selectivity across metal catalysts. Additionally, DFT studies on FUR adsorption on Ni(111) and Ni(211) confirm that adsorption geometries and hydrogenation pathways are step-site dependent. These findings underscore the importance of step-site coordination in catalysis, reinforcing the use of high-index surfaces as models for investigating how unique coordination environments influence reactivity and selectivity.^36,37^

This study is the first to explore FUR reactivity on high-index Ni(119) using both experimental and theoretical approaches, examining clean and carbon-passivated surfaces in presence and absence of hydrogen. Using a wide range of in situ techniques such as photoelectron spectroscopy (XPS/UPS), Kelvin probe, and thermal desorption spectroscopy (TPD), complemented by density functional theory (DFT) calculations we analyzed the electronic properties, energetics, adsorption geometry, and desorption behavior. Our results reveal a strong interplay between electronic and geometric effects and structure-dependent reactivity relationships. Notably, we report for the first time the self-hydrogenation of FUR to 2MF on clean Ni(119), a reaction that is not observed on the carbon-passivated surface. The surface steps were found to act as a hydrogen transfer pumps (HTPs), facilitating hydrogenation reactions. Both surface steps and reactively formed nickel carbides were found to hold the key in directing reaction selectivity and modulating overall surface reactivity. Reaction selectivity toward furan correlates with the (001) terrace surface, while selectivity toward 2MF is associated with the presence of steps. By extending the hydrogen transfer pump mechanism from Ni(119) to Ni nanoparticle catalysts, this study bridges fundamental surface science with practical catalysis, establishing a direct correlation between model surface activity and real-world catalysts, revealing how coordination governs selective adsorption and active site preferences. These findings advance the understanding of furfural reactions on complex Ni surfaces, with significant implications for efficient and selective biomass conversion technologies.

Figures 1a–d present the desorption behavior and reactivity of FUR and its derivatives such as FAL, 2MF, and furan, on a Ni(119) stepped surface, using thermal desorption spectroscopy (TDS). The experiments were conducted by exposing 0.8 monolayers (ML) of each molecule to the surface at 170 K and heating at a rate of 1 K s^–1^. Each molecule clearly exhibits distinct desorption characteristics. FUR (Figure 1a) desorbs predominantly at 225 K, while at higher temperatures, it undergoes decarbonylation to form furan (270 K). Notably, hydrogenation products like 2MF are detected (*m*/*z* = 82), indicating the self-hydrogenation of FUR. In contrast, the TPD spectra of furan (Figure 1b) exhibit lower reactivity, with the molecules desorbing mainly unreacted at 220 K with minimal formation of decomposition products, such as CO and H_2_, due to limited ring cleavage. Similarly, unreacted 2MF (Figure 1c) desorbs at a lower temperature (190 K), while higher temperatures promote the formation of ring-opening products and 2-methyltetrahydrofuran (2-MeTHF). FAL exhibits a complex desorption profile, predominantly yielding 2MF and furan, with distinct desorption peaks observed at 225 K for FAL (Figure 1d), 295 K for 2MF, and 310 K for furan, highlighting its intricate reactivity.

**Figure 1:**
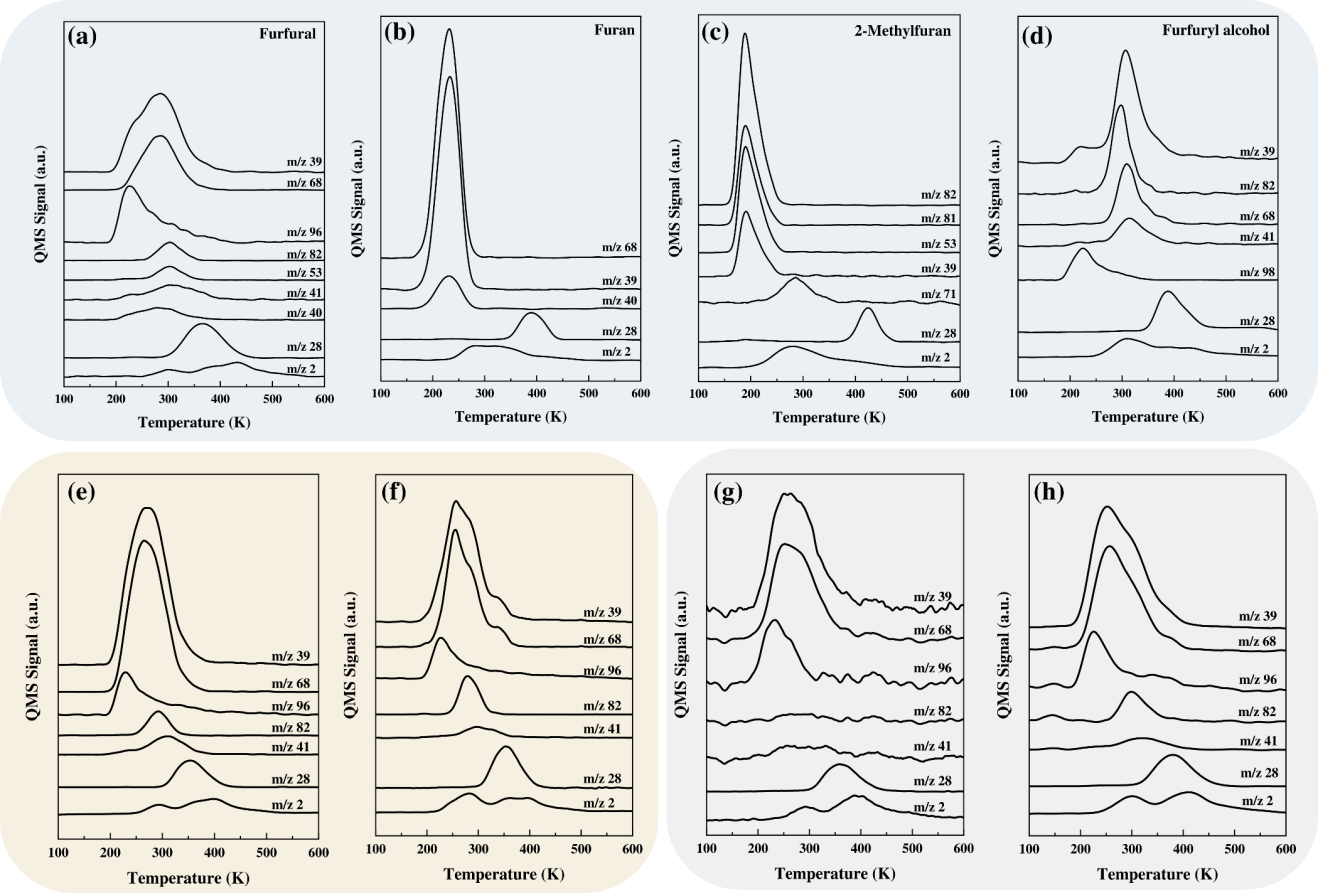
TPD spectra for (a) FUR, (b) furan, (c) FAL, and (d) 2MF following the adsorption of 0.8 ML on the Ni(119) surface at 170 K. Panels (e) and (f) show TPD spectra for 0.5 ML FUR on a clean Ni(119) surface and for coadsorbed FUR (0.5 ML) and H_2_ (0.5 ML), respectively. Panels (g) and (h) illustrate TPD spectra from the carbon-passivated Ni(119) surfaces, with panel (g) showing the desorption of 0.5 ML FUR and panel (h) the desorption of coadsorbed FUR (0.5 ML) and H_2_ (0.5 ML). All experiments were conducted at 170 K with a heating rate of 1 K s^–1^. Detected species included FUR (*m*/*z*: 39, 96), tetrahydrofuran (*m*/*z*: 39, 72), furan (*m*/*z*: 39, 68), 2MF (*m*/*z*: 39, 53, 82), FAL (*m*/*z*: 39, 98), propene (*m*/*z*: 39, 41), H_2_ (*m*/*z*: 2), and CO (*m*/*z*: 28).

The self-hydrogenation of unsaturated organic molecules on Ni catalysts, is significantly influenced by the coordination number of nickel surface atoms.^38^ Studies on acetylene,^38,39^ ethylene,^40^ ethene^41^ and acrolein^42−44^ across various metal substrates suggest that self-hydrogenation depends on nickel atom coordination. On flat nickel surfaces, the dissociation of C–C bonds occurs at lower temperatures, compared to C–H bond dissociation, whereas, on stepped surfaces, C–H bond activation is more favorable. This behavior likely contributes to the formation of hydrogenated species during the desorption of FUR from stepped Ni surfaces. Additionally, FUR and FAL produce mostly furan and CO from Ni(119) surface, indicating that the surface primarily activates decarbonylation reaction pathways. Notably, for FAL, decarbonylation occurs after its reduction to FUR, underscoring the significant impact of surface steps on the reaction mechanism. The observation that surface steps may act as hydrogen transfer pumps (HTPs) highlights their role in facilitating hydrogenation by dragging and releasing protons during the reaction. To further investigate the hydrogen transfer mechanism, future studies could employ isotope labeling experiments (e.g., H_2_/D_2_ exchange studies) and potentially scanning tunneling microscopy (STM) to directly track hydrogen migration pathways and validate the role of step sites in hydrogen transport.^45^

Based on the results presented in Figures 1a–d, FUR exhibits a high conversion rate (∼65%) with a selectivity of ∼75% toward furan, ∼8% toward 2MF, and ∼13% toward carbon (see Section S1 in the Supporting Information for TDS spectra quantification). Across the studied molecules, FAL achieves the highest overall conversion (∼78%), with substantial selectivity toward 2MF (∼46%) and furan (∼36%). In contrast, 2MF and furan show lower conversions (∼20% and ∼10%, respectively) and different product distributions, suggesting that their reactivity is heavily influenced by the presence of the functional groups. This finding highlights the crucial role of surface structure in catalytic reactions involving complex molecules like those derived from lignocellulosic biomass.

The results presented thus far outline the interaction of FUR and its primary byproducts with the Ni(119) surface. However, a detailed mechanistic understanding of the hydrogenation processes occurring on this surface requires further study. Figures 1e and 1f show the TDS spectra for 0.5 ML of FUR adsorbed on both pristine and carbon-passivated Ni(119) surfaces, in the presence and absence of preadsorbed hydrogen (H_a_). Carbon passivation of the Ni(119) surface results from a prior TPD cycle, during which hydrocarbons decomposed thermally, leaving a residual carbon layer. On both pristine and carbon-passivated Ni(119) surfaces, the desorption of FUR (*m*/*z* 96) and furan (*m*/*z* 68) occurs at 220 and 270 K, respectively. However, the carbon-passivated surface demonstrates an enhanced production of furan, underscoring the impact of residual carbon in modifying reaction selectivity. This behavior indicates that residual carbon inhibits self-hydrogenation while promoting decarbonylation. The selective inhibition of self-hydrogenation can be attributed to carbon deposition along monatomic steps on the Ni(119) surface. Carbon deposition selectively blocks active sites, preventing the activation of C–H bonds necessary for self-hydrogenation and shifting the reaction toward exclusive decarbonylation pathways. This effect with previous findings by Blakely et al.,^46^ which demonstrated that carbon preferentially deposits along monatomic steps, altering catalytic behavior. The lower recombinative desorption of H_2_ on the carbon-passivated surface compared to that on the pristine Ni(119) surface (see Section S2 in the Supporting Information for more details) further supports this hypothesis.

The TDS spectra in Figures 1f–h also illustrate that the desorption temperature of FUR remains constant at 220 K on clean Ni(119), irrespective of H_a_, due to the dissociative adsorption of H_2_. In contrast, the desorption profile of furan changes significantly in the presence of H_a_, with three distinct peaks observed at 270, 300, and 350 K. The interpretation of these distinct furan desorption features requires careful consideration, as similar catalytic behavior is not well-documented in the existing literature (a detailed discussion of these results is provided in Figures S6 and S7). The additional peaks suggest complex surface interactions and reaction pathways facilitated by H_a_. Interestingly, the formation of 2MF is consistently observed at 285 K on both pristine and carbon-passivated surfaces, regardless of the presence of H_a_. This observation implies that the presence of carbon does not significantly impact 2MF formation, highlighting the robustness of this reaction pathway on Ni(100) terraces in the presence of H_a_. The latter, underscores the complexity of surface interactions and reaction pathways in the presence of hydrogen, emphasizing the nuanced and dynamic nature of surface chemistry under varying chemical environments.

The conversion of FUR across a range of surface coverages (0.25–2 ML) is presented in Figure 2. On the pristine surface, the selectivity to 2MF at low FUR coverages, increases 2- to 3-fold, compared to the absence of hydrogen, indicating that the presence of H_a_ enhances the formation of hydrogenolysis products. However, as the concentration of FUR increases (resulting in a corresponding decrease in H_a_), 2MF selectivity decreases while furan selectivity rises, indicating a direct correlation between surface hydrogen concentration and product selectivity (Figure 2g). Conversely, on the carbon-passivated surface, the presence of H_a_ slightly reduces 2MF selectivity with minimal overall effect on product distribution. The reduced selectivity, compared to the pristine surface, highlights the surface poisoning and the concurrent inhibition of self-hydrogenation reactions (Figure S3). The site- and coverage-dependent adsorption selectivity presented in this study inherently captures the dynamic interactions between hydrogen and FUR on Ni(119). Specifically, the role of step sites as HTPs dynamically modulates hydrogen transfer, influencing selectivity toward 2MF. Similar to Wang et al.,^47^ who used DFT-based microkinetic modeling to study coverage-induced conformational changes on Pd(111), this study shows that, on Ni(119), selectivity is dynamically modulated by the conformational states governed by hydrogen coverage.

**Figure 2:**
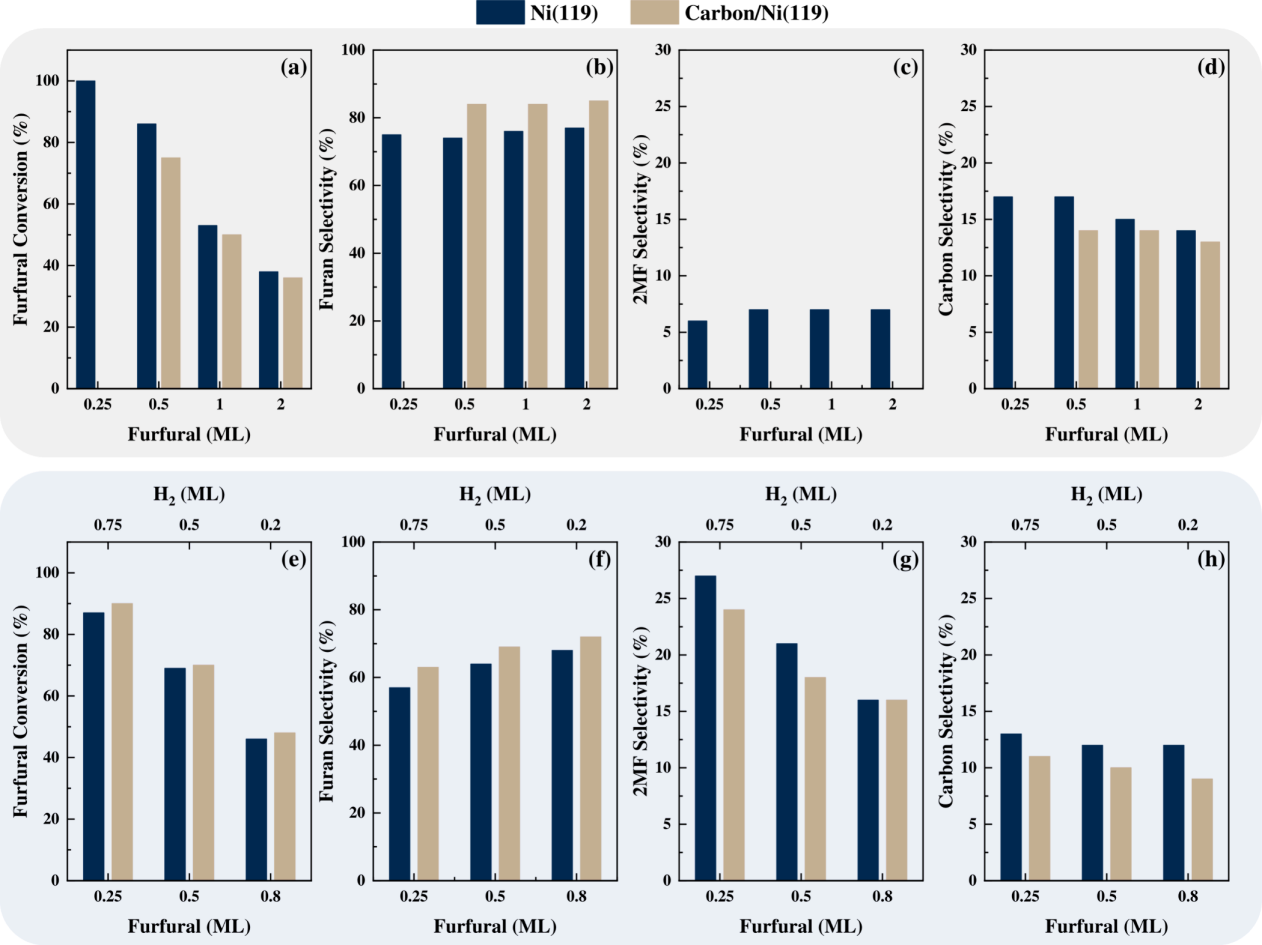
Bar charts illustrate the impact of varying FUR (0.25–2 ML) and hydrogen (0.2–0.8 ML) concentrations on product conversion and selectivity. Panels (a)–(d) present FUR conversion, furan selectivity, 2MF selectivity, and carbon selectivity, respectively, as functions of FUR concentration. Similarly, panels (e)–(h) depict these metrics under different hydrogen concentrations. The results compare the behavior of Ni(119) and carbon/Ni(119) surfaces.

Our findings provide a detailed mechanistic framework for FUR conversion on Ni(119), illustrated in Scheme 1. Initially, chemisorbed FUR undergoes self-hydrogenation and decarbonylation, producing FAL and furan, respectively. While furan desorbs at 270 K, the strongly bound FAL remains on the Ni(119) surface, allowing it to undergo further hydrodeoxygenation and decarbonylation to produce 2MF and additional furan. The desorption profiles of FUR and FAL show that 2MF desorbs at 280 K, as illustrated in Figure 1 and Figures S6 and S7. Notably, FAL remains a key surface-bound intermediate, transforming into 2MF on the Ni(119) surface. This transformation involves hydrogen transfer, facilitated either by surface-generated hydrogen atoms upon FUR C–H bond cleavage or by intramolecular transfer from hydroxyl groups within FUR. The role of FAL as an intermediate is evident from the higher desorption temperature of furan at 300 K, compared to its desorption at 270 K when it forms directly from FUR. This temperature difference indicates that FAL undergoes a slower, stepwise decarbonylation with a higher energy barrier for C=O bond cleavage, requiring progressively more energy until furan desorbs at 300 K. Furthermore, the absence of a high-temperature furan desorption peak (>300 K) during FUR TDS without H_2_ indicates lower selectivity toward 2MF. Conversely, the presence of H_2_ enhances 2MF formation, underscoring the role of surface hydrogen (H_a_) in driving the reaction and stabilizing FAL as a surface-bound intermediate.

**Scheme 1:**
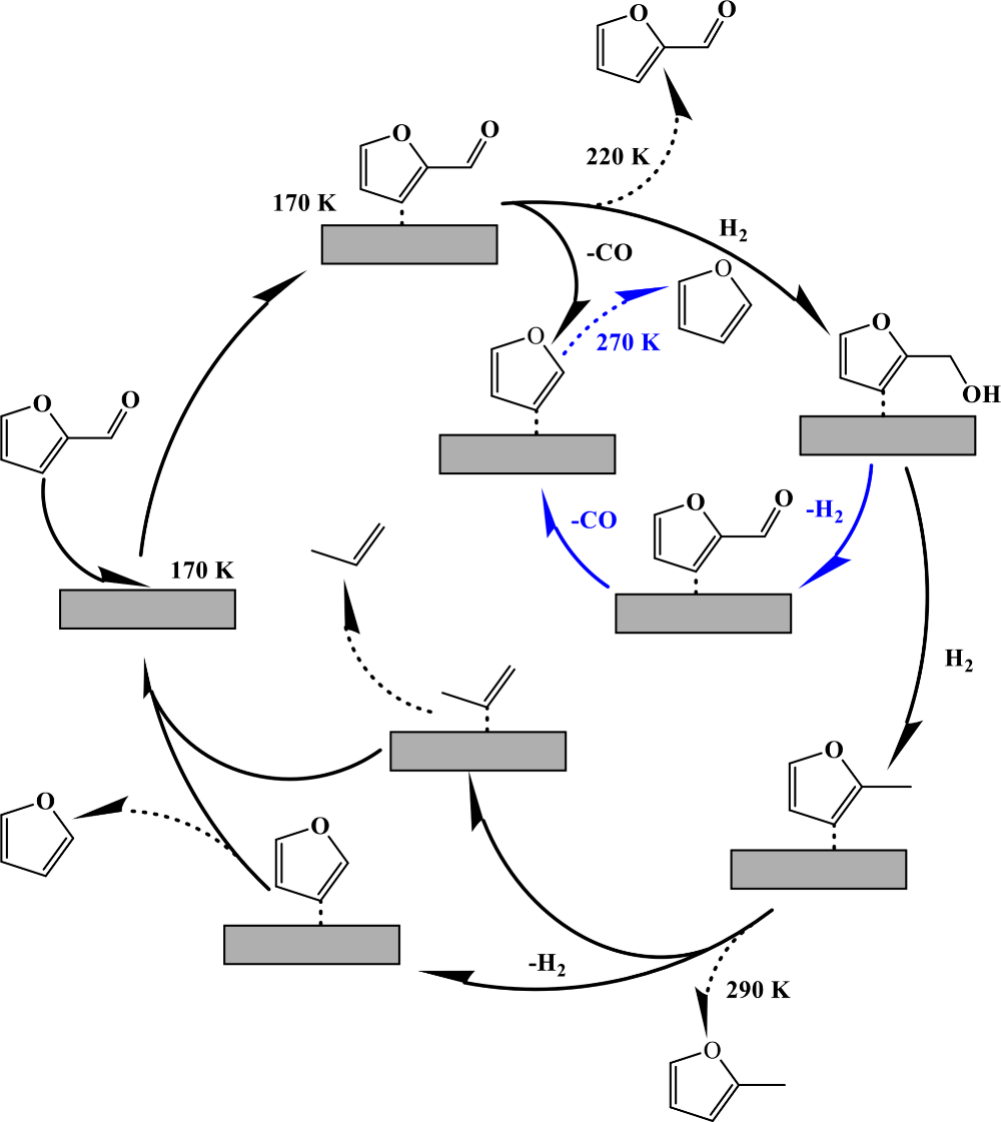
Schematic of the Proposed Reaction Pathway for FUR Conversion on the Ni(119) Surface, Highlighting Key Intermediates and Temperature Conditions (170, 220, 270, and 290 K) Observed in TDS Experiments FAL acts as a surface-bound intermediate, transforming into 2MF without desorption. This transformation involves hydrogen transfer from surface-generated hydrogen or intramolecularly from hydroxyl groups. The inner blue cycle represents proposed intermediate steps of surface-bound reactions and is not evident as a gas-phase product in the reaction scheme.

A detailed spectroscopic analysis of FUR adsorption on Ni(119) was performed to establish correlations between thermal desorption profiles (TPD/TPR) and the reactive transformations of FUR. The surface was initially exposed to 2 ML of FUR at 175 K, followed by controlled annealing to the target temperature at a rate of 1 K s^–1^, and subsequent rapid cooling to 170 K. This process is illustrated in Figures 3a and 3b, which shows the corresponding changes in the C 1s and O 1s regions of the XPS spectra. Detailed deconvolution of the spectra is presented in Section S3 and Figure S8 in the Supporting Information. Initially, FUR formed a multilayer film, transitioning to a submonolayer state at 205 K, as indicated by the reduction in C 1s and O 1s peak intensities (Figure S9). This transition, with a 1:1:0.5 (C_1_–C_2_:C_3_–O_1_–C_4_:C_5_–O_2_) peak ratio, suggests limited decomposition up to 205 K (see Table S3). At 225 K, the R–C–O peak (287.5 eV) decreased significantly, signaling FUR desorption and decarbonylation, forming surface-adsorbed furan and CO. The shifts in C 1s peaks at 285.5 and 287.5 eV confirmed CO at linear and bridging sites (Figure S10), with CO desorbing around 400 K, consistent with chemisorbed CO on Ni(119).^48^ Further heating to 300 K led to FUR decomposition, and the formation of furan and C_*x*_H_*y*_ intermediates. The stability of the CO-related peaks between 225 and 300 K, followed by their disappearance, corroborated TPD trends.^18^ The O 1s spectra (Figure 3b) further supported these findings, showing a decline in aromatic oxygen species and an increase in the C–O peak, indicative of decarbonylation and CO evolution.

**Figure 3:**
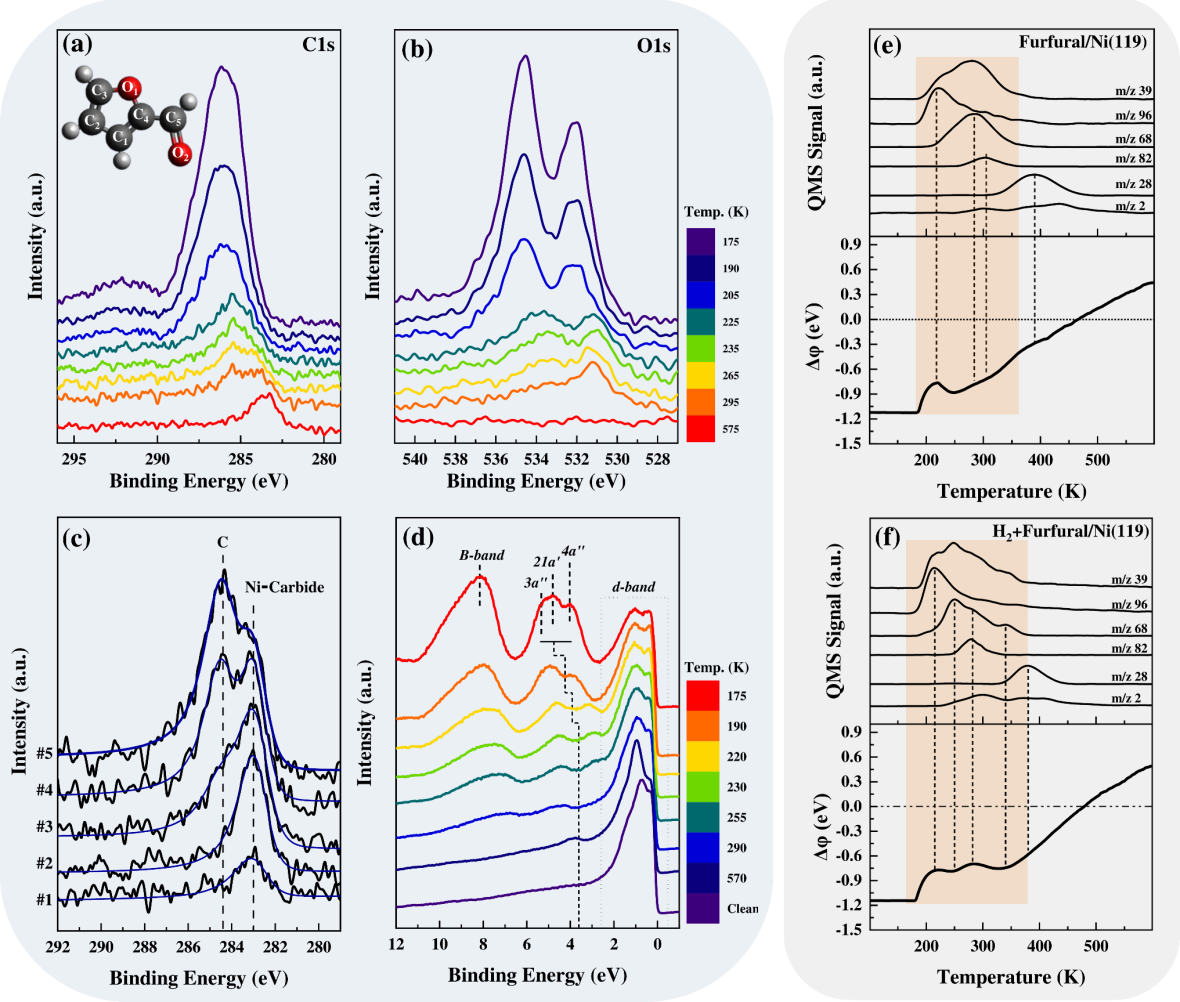
(a) Temperature-dependent C 1s XPS spectra from 175 to 575 K for an initial FUR coverage of 2 ML. (b) XPS spectra of the O 1s region under the same temperature conditions, revealing the chemical state variations of oxygen. (c) Detailed XPS spectra of the C 1s region for five cycles of FUR adsorption (1 ML) at 180 K, followed by rapid heating to 550 K, illustrating the dynamic adsorption–desorption process. (d) UPS HeI spectra (21.2 eV) for an initial 1 ML coverage of FUR on the Ni(119) surface at 175 K, displayed as a function of annealing temperature to elucidate electronic structure changes. (e) Work function change (Δϕ) profiles during the thermal desorption of ∼1 ML FUR from a clean Ni(119) surface, coupled with corresponding TPD/TPR spectra (heating rate = 1 K s^–1^). (f) Work function change (Δϕ) and TPD/TPR spectra for 0.8 ML FUR in the presence of a preadsorbed 0.2 ML hydrogen on the Ni(119) surface, highlighting the influence of preadsorbed hydrogen on FUR desorption dynamics.

A key finding was the formation of surface carbides as the C 1s peak shifted to 283.5 eV at 575 K (Figure 3c), indicative of carbide growth at the monatomic step edges of Ni(119), which act as catalytic hot spots.^49^ Carbide formation was limited to a coverage of 0.2 ML, aligning well with the number of steps present on the surface (Figure S11). The step-selective carbide formation mirrors the behavior observed in metastable nickel carbide systems, where low-coordination sites preferentially stabilize carbon species as reported by Bayer et al.^50^ using in-situ Raman and XPS on Ni_3_C nanoparticles. After five FUR adsorption–desorption cycles, a C 1s peak at 284.1 eV emerged, indicating the development of graphitic carbon following the completion of carbide growth. This transition is driven by the saturation of step sites, promoting carbon–carbon coupling and graphitization. The progression from step-selective carbide formation to graphitic carbon growth underscores the role of step edges in carbon species formation and highlights their relevance to the observed catalytic activity.

The conversion of FUR on the Ni(119) surface was further examined through UPS HeI measurements at normal emission (θ = 0°) following a 1 ML exposure at 175 K, as presented in Figure 3d. The UPS spectra revealed distinct emission bands for FUR and other formed surface products (see Section S3 and Figures S12 and S13 in the Supporting Information). It is worth mentioning that the condensed phase of FUR displays two distinct emission bands within the energy ranges of 3–7 eV and 7–12 eV. Specifically, the 3–7 eV band features three peaks associated with molecular orbitals of symmetries 4*a*″ (π), 21*a*′ (σ), and 3*a*″ (π), while the 7–12 eV band exhibits a broad feature due to state superposition.^18^ At temperatures between 220 K and 260 K, the spectra align well with those of adsorbed furan, confirming its formation. The presence of 2MF peaks cannot be ruled out due to spectral similarities with furan. CO detection is evident in the 6–8 eV range (centered at 7 eV), as shown in Figure S13, presenting HeI and HeII spectra during CO adsorption on Ni(119). The study reveals a significant cross-section difference at 21.2 eV versus 40.8 eV, making CO states (5σ, 4σ, and 1π) indistinct, except for a clear 7 eV peak in the HeI spectrum, representing the 1π state. These results confirm that adsorbed FUR primarily converts to furan and CO, remaining on the surface up to 300 K before desorbing or decomposing into other carbon species. At 570 K, a 4 eV peak emerges, indicating carbide formation, consistent with XPS studies by Paolucci et al.,^51^ identifying this peak as characteristic of carbide π states.

In-situ vibrating capacitance Kelvin probe (VCKP) measurements during FUR desorption (Figures 3e and 3f) provided real-time insights into the system dynamics, capturing gas-phase product formation and work function changes due to desorption and surface-bound species evolution. Initially, the work function difference (Δϕ) exhibited a peak at 220 K and a trough at 250 K (Figure 3e), which was consistent with FUR desorption and furan formation, as also confirmed by TPD. This behavior reflects the formation of an adsorbate-induced dipole layer, where FUR, acting as an electron acceptor, withdraws electron density from the Ni(119) surface. Above 300 K, CO became the predominant surface species, leading to a steady increase in Δϕ, again supported by the TPD data showing CO desorption around 400 K. The presence of preadsorbed hydrogen (Figure 3f) introduced distinct peaks and troughs in the Δϕ profile, indicating the formation of hydrogenated intermediates, such as surface-bound FAL derivatives. This resulted in a new local minimum in Δϕ. As FAL transformed into 2MF and subsequently desorbed, Δϕ increased, resembling the behavior observed on the pristine surface. Furthermore, nickel carbide becomes predominant above 450 K, leading to a further increase in Δϕ, consistent with prior studies on Ni surfaces.^52,53^ Above 700 K, the work function declines, reaching near zero beyond 850 K, likely due to carbon migration into the crystal matrix (Figure S14). Overall, the adsorption, desorption, and transformation of FUR on Ni(119) reveal temperature-dependent interactions and the formation of intermediate species, providing key insights into the surface chemistry and electronic structure changes during FUR surface transformation.

Utilizing UPS and DFT, the adsorption geometry and the surface-molecule interactions were investigated. Angle-dependent UPS experiments were conducted at 175 K for FUR coverages ranging from 0 to 1 ML on a clean Ni(119). Figure 4 presents the UPS spectra at polar angles (θ) ranging from 0° to 45°. Notably, the polarization components along the molecule’s *z*-direction, characterized by their π nature, diminish as the incident light angle shifts from 0° to 90°. This indicates that, for spectra acquired at an incident radiation angle of 0°, emissions from polarized molecular orbitals along the *z*-axis are not expected to produce distinct peaks due to selection rules derived from Fermi’s golden rule.^54^

**Figure 4:**
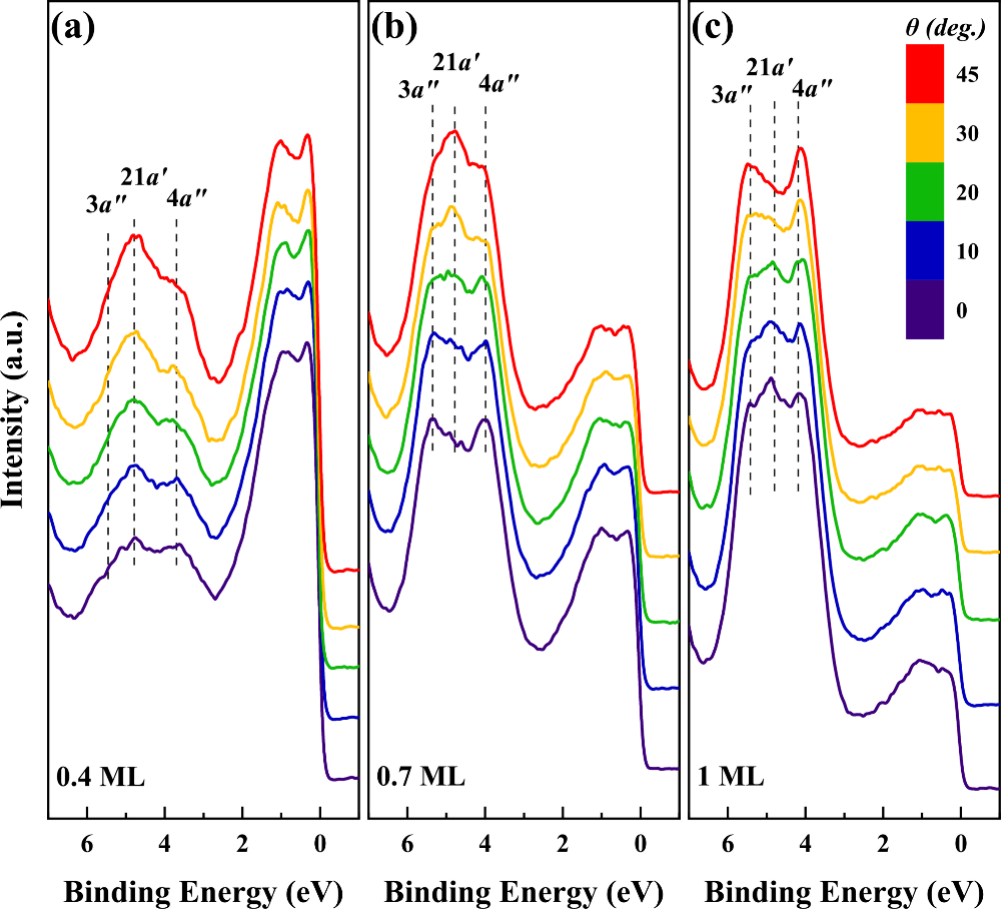
HeI ultraviolet photoelectron spectroscopy (UPS) spectra at 21.2 eV for the Ni(119) surface upon (a) 0.4 ML of FUR, (b) 0.7 ML of FUR, and (c) 1 ML of FUR measured at 170 K. The spectra illustrate variations as a function of the polar angle θ, spanning from 0° to 45°.

The results reveal that at low coverages (0.4 and 0.7 ML), FUR molecules predominantly align nearly parallel to the surface. This is evidenced by the spectral peaks corresponding to the highest occupied molecular orbitals (HOMOs), specifically the 4*a*″, 21*a*′, and 3*a*″ states, which appear prominently at 3.8, 4.8, and 5.5 eV, respectively, at a polar angle of 0°. Changes in the polar angle (θ) lead to significant intensity variations in these peaks, highlighting the polarization of molecular orbitals along the *z*-axis. More specifically, increasing the polar angle reduces the intensities of the 4*a*″ and 3*a*″ peaks while enhancing the 21*a*′ peak. These variations indicate a nearly parallel molecular alignment at lower coverages, attributed to the polarization of the *a*″ orbitals along the *z*-axis, normal to the molecular plane, suppressing emission from these orbitals. At 1 ML coverage, the UPS spectra show a clear shift, indicating a change in adsorption geometry. Specifically, the reduction of the 21*a*′ peak and the increase of the 3*a*″ and 4*a*″ peaks, with increasing polar angles, suggest a shift from a near-parallel (η^4^ – η^6^) to a perpendicular (η^1^) configuration, consistent with previous studies,^16,18^ which suggests that as surface coverage increases, spectral shifts suggest a transition from parallel to more-inclined molecular orientations.

Furthermore, we employed DFT calculations to elucidate the adsorption mechanism of FUR on the stepped surface, by examining how both electronic and geometric contributions influence the adsorption configurations, energetics, and ultimately, catalytic activity and selectivity. This approach offers fundamental insights into adsorption energetics and conformational changes without relying on transition state theory (TST) rate calculations. Our strategy is supported by microkinetic modeling and DFT studies,^55,56^ which highlight the crucial impact of surface coordination and coverage on product distribution, emphasizing the importance of adsorption configurations. Moreover, the computational results presented in Figure 5 (see Figure S15, as well as Sections S3 and S4, in the Supporting Information, for more details) were crucial for interpreting the spectroscopic and spectrometric data. The analysis focused on three reactive regions of the surface: the upper step edge (S), terrace (T), and underneath step edge (U). Figure 5 illustrates FUR adsorption at these sites, labeled S_1_, T_1_, and U_1_, revealing a clear preference for the molecule to adsorb in a planar orientation parallel to the metal surface, termed “south”, with the polarization vector pointing toward the  direction. The same methodology applies to the remaining FUR adsorption configurations, as outlined in the Supporting Information.

**Figure 5:**
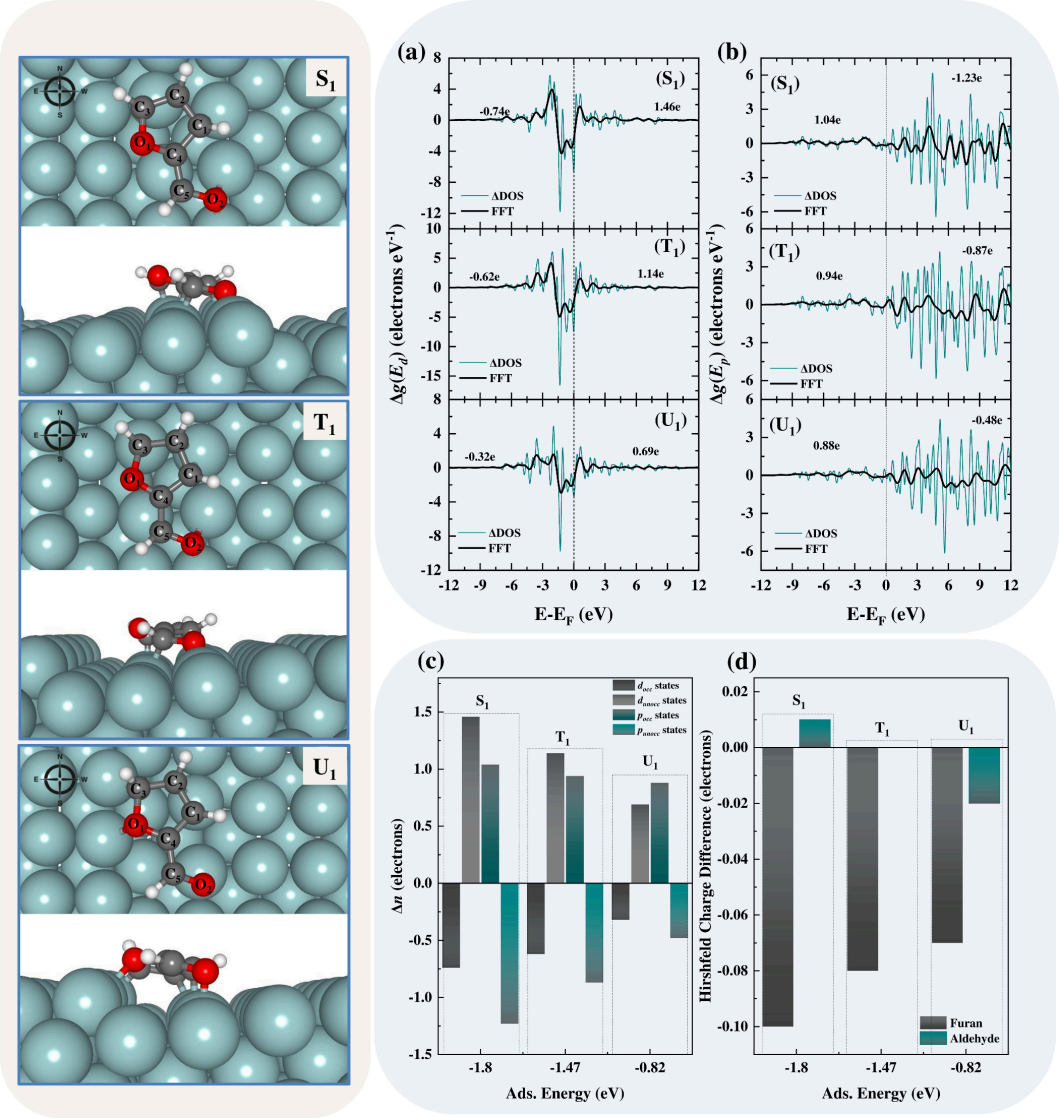
(Left) Adsorption configurations of FUR on a Ni(119) stepped surface: S_1_ (step edge), T_1_ (on the terrace), and U_1_ (underneath step edge). Changes in the density of states (Δ*g*(*E*)) for (a) *d* states and (b) *p* states and subtracted from the baseline of the pure Ni surface. (c) Δ*n*_occ_ and Δ*n*_unocc_ for *d* and *p* states, split by states below and above the Fermi level, indicating occupied and unoccupied states. (d) Hirshfeld charge analysis showing electron gain or loss in FUR, compared to its isolated molecular state.

FUR preferentially adsorbs at the top steps (S_1_) with a binding energy of −1.80 eV, due to increased electrophilicity and a less-coordinated environment that enhances interactions with FUR functional groups (see Table S4). At S_1_, FUR exhibits an η^6^-π(C_1_C_4_)-π(C_2_C_3_)-diσ(C_5_O_2_–NiNi) coordination, enabling multiple (diσ) bonds and π interactions. These interactions are critical, as π bonds facilitate electron delocalization, strengthening the interaction with the surface through resonance stabilization, while diσ bonds involve localized electron sharing between the molecule and metal atoms, enhancing stability. Moreover, the inclusion of multiple diσ interactions, particularly from the aldehyde group, increases the number of bonding sites, contributing to stronger adsorption. At the terrace site (T_1_), FUR adsorption energy is −1.48 eV with an η^5^-π(C_1_C_2_)-σ(C_3_)-diσ(C_5_O_2_–NiNi) coordination. Despite adequate bonding, fewer π interactions compared to S_1_ suggest suboptimal electron delocalization. The lower step edge (U_1_) exhibits the weakest adsorption energy of −0.82 eV, with an η^5^-π(C_1_C_2_)-diσ(C_3_O_1_)-σ(O_2_) coordination. Here, extensive electron delocalization is hindered due to Pauli repulsion,^57^ which arises from overlapping electron-rich orbitals. This repulsion destabilizes adsorption, despite the high electron density at the lower step.

Root-mean-square deviation (RMSD) values for FUR are similar across different configurations, indicating comparable molecular deformation upon adsorption, while surface deviation increases with higher adsorption energy and bonding complexity (refer to Section S6 and Table S4 in the Supporting Information for more details). Moreover, a side-view analysis of the adsorption configurations shown in Figure 5 reveals significant structural deformation upon adsorption. Clearly, the H and O atoms in the furan ring tilt away from the surface plane, while the Ni(119) surface bonding atoms undergo relaxation and move upward, initially shifting toward the molecule. Adsorption induces significant structural distortions in FUR, especially in the C_5_–O_2_ and C_3_–O_1_ bonds, which elongate by up to 12.6% and 11.38%, respectively, enhancing potential bond cleavage potential for catalysis (Table S5). This strong interaction through both the furan ring and the aldehyde group, likely shifts FUR from a pure aromatic planar configuration to a distorted state indicative of sp^2^ to sp^3^ rehybridization, with the greatest deformation at the S_1_ site, which correlates with the highest adsorption energy.

In order to enhance our understanding of the complex interaction of FUR with the stepped surface, we conducted differential density of states (Δ*g*(*E*)) calculations of the Ni(119) termination layer, as well as Hirshfeld charge analysis of the FUR (see Section S5 in the Supporting Information for more details). Analysis of Δ*g*(*E*) for the *d* and *p* states across FUR adsorption sites reveals that occupied *d* states exhibit a progressive electron loss from U_1_ (−0.320) to S_1_ (−0.740), suggesting stronger electron withdrawal and interaction at sites with higher adsorption energies, indicative of intense back-bonding or charge-transfer mechanisms. Conversely, unoccupied *d* states show a decrease in electron density from S_1_ (1.460) to U_1_ (0.690), reflecting effective electron utilization in bonding at sites with stronger adsorption. For *p* states, the trend in occupied states indicates an increase in electron density when moving from energetically favorable to less favorable sites (S_1_ to U_1_), whereas unoccupied *p* states exhibit a progressive electron loss, decreasing from U_1_ (−0.480) to S_1_ (−1.230). This pattern suggests significant electron redistribution within the *p* orbitals, likely supporting bonding interactions, such as π-bonding, which contribute to the stabilization of adsorption at these sites. The interplay between the occupied and unoccupied states in the *d* and *p* orbitals underscores a complex pattern of electron allocation and bonding at different adsorption sites. For the *d* orbitals, stronger adsorption correlates with greater electron depletion from the occupied states and more pronounced electron utilization in the unoccupied states, reinforcing the adsorption bonds. For the *p* orbitals, the behavior suggests a complementary pattern of electron gain in the occupied states and loss in the unoccupied states, which influences the overall stability and effectiveness of the adsorption process.

Hirshfeld charge difference analysis (Figures 5c and 5d) provides insights into electron redistribution upon FUR adsorption on nickel surfaces. Across the adsorption energies examined, FUR consistently exhibits a net electron gain (approximately −0.090), indicating enhanced electrostatic and chemical interactions with the nickel surface. Specifically, the furan ring shows increased electron density in stronger adsorption scenarios (e.g., −0.100 at S_1_), suggesting significant π-electron backbonding with the nickel as mentioned before. Conversely, the aldehyde group exhibits variable electron dynamics, gaining electrons at higher adsorption energies (−0.010 at S_1_) and losing at lower (−0.020 at U_1_). This behavior highlights the role of aldehyde in adapting to different electronic environments, influencing FUR orientation and reactivity on the surface, reflecting its adaptability to different chemical environments.

Overall, the top step edge configuration exhibits the most complex and stable bonding, including diσ and π interactions across multiple atoms. This optimal configuration match, where the molecular orientation and bonding configuration align well with electron availability and surface geometry, not only influences the stability of the adsorbed molecule but also affects its reactivity. Different adsorption sites can activate different parts of the molecule, affecting catalytic activity or reaction pathways. Although kinetic barriers are not explicitly calculated in this study, the findings align with the literature, which suggests that Langmuir–Hinshelwood kinetics best describe gas-phase FUR hydrogenation. This highlights the importance of surface site availability and adsorption configurations in optimizing catalytic performance.^58^ Such insights underscore the complexity of catalyst surface design, where both geometric and electronic properties must be tailored to optimize reactivity and selectivity for desired reactions. Specifically, our results suggest that controlling surface step density will have a pronounced effect on both the activity and selectivity of the reaction.

Building on the preceding discussion, a critical step in the rational design of efficient catalysts for FUR conversion is to decode how selective adsorption and active site configurations govern the reaction pathways. A central question is whether model catalyst studies can reliably mimic the specific active sites that drive selectivity in practical nanoparticle catalysts. To address this, we examine the coordination environments of Ni atoms on a model Ni(119) surface by analyzing the coordination number (*cn*) and the generalized coordination number .^59^ These descriptors play a crucial role in establishing connections between model surfaces and real catalysts, serving as a conceptual bridge between idealized atomic configurations and the morphologies of industrially relevant nanoparticles.

Scheme 2 illustrates how surface Ni atoms on Ni(119) correlate with spherical Ni nanoparticles of various diameters (1–2.5 nm) by matching atoms with similar *cn* and  values (within ±5%), based on the methodology detailed in Section S7 in the Supporting Information and previous studies.^60^ This matching approach is essential for transferring fundamental insights from model systems to nanoparticles, where controlling the distribution of coordination environments enables precise modulation of catalytic selectivity and efficiency.^59^ For instance, Ni surface sites characterized by *cn* = 7, which are found on stepped Ni surfaces, exhibit intrinsic selectivity toward 2MF. In contrast, Ni(001) facets with *cn* = 8 favor the formation of furan, and Ni(111) facets promote propene formation.^24^ Our analysis indicates that particles smaller than approximately 1.5 nm lack such *cn* = 7 sites, whereas 2 nm particles provide coordination environments analogous to the stepped surface, and 2.5 nm particles encompass the full range of active site types identified on Ni(119). These findings highlight a direct link between particle size, site distribution, and reaction selectivity. Moreover, pyramidal Ni particles possess edges that behave similarly to stepped sites, thereby enhancing hydrogenation selectivity,^61^ while their planar facets, analogous to (111) surfaces, readily cleave the furanic ring of FUR. Likewise, cubic Ni particles, which mimic (001) facets, facilitate selective cleavage between the furan ring and the aldehyde group.^62^ Collectively, this underscores that nanoparticle shape and size—by dictating the available coordination environments—strongly modulate catalytic behavior.^29,63^ These facet-specific behavior provide crucial insights into the catalytic roles of different coordination environments and their potential under practical conditions, such as in hydrogen-rich environments where these activation steps can be followed by further hydrogenation. Understanding site-selectivity relationships enables the rational design of Ni-based catalysts, offering a pathway to optimize selectivity and efficiency in biomass conversion processes.

**Scheme 2:**
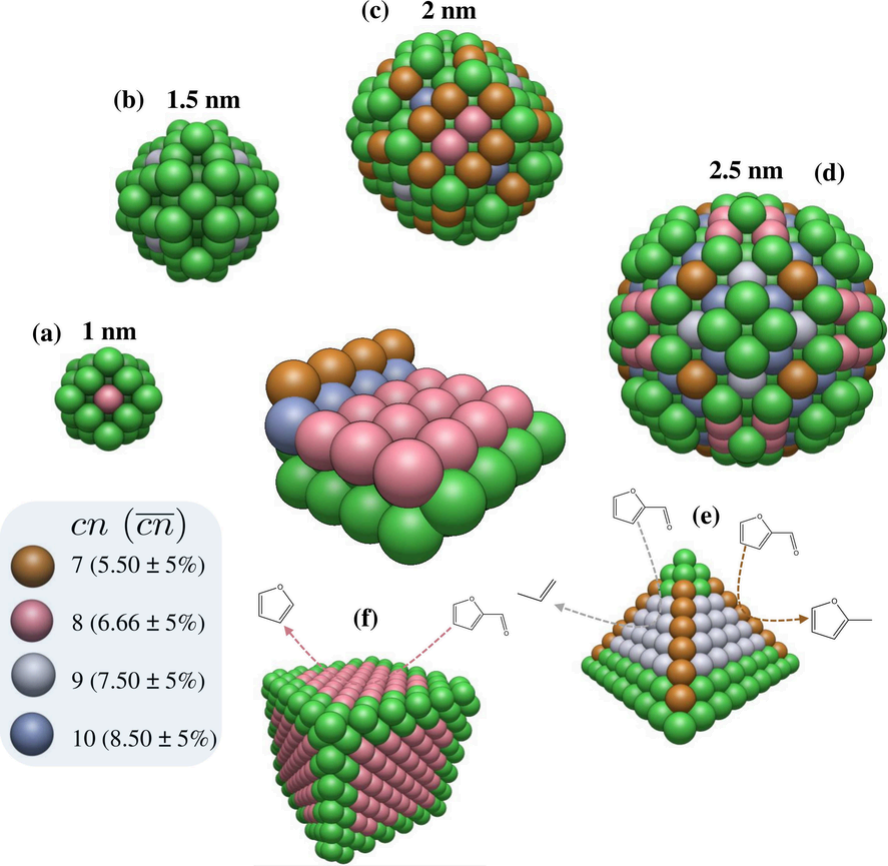
Visualization of Coordination Numbers (*cn*) and Generalized Coordination Numbers  of Ni Atoms on Ni(119) Surfaces and Nanoparticles of Varying Diameters (1–2.5 nm) and Shapes, Illustrating the Distribution of Active Sites Relevant for Catalytic Reactions

To conclude, this study is the first to systematically investigate FUR reactivity on high-index Ni(119) surfaces using combined experimental and theoretical approaches. Our findings show that the unique topology of the Ni(119) surface promotes self-hydrogenation of FUR to 2MF while suppressing decarbonylation—the selectivity driven by step sites acting as ”hydrogen transfer pumps”. These sites extract hydrogen from adsorbed FUR and facilitate its diffusion to terrace-bound molecules, enhancing selective hydrogenation—a catalytic behavior absent on flat or carbon-passivated surfaces, underscoring the essential role of step sites in guiding reaction pathways. The rapidly formed carbides, shift the reaction pathway toward decarbonylation and restrict self-hydrogenation, highlighting the critical role of surface cleanliness and hydrogen availability in optimizing catalytic performance.

Mechanistically, pristine surfaces with preadsorbed hydrogen favor hydrogenolysis, whereas carbon-passivated surfaces reduce selectivity due to the carbon inhibitory effect on self-hydrogenation. On Ni(119), furfural aligns parallel to the surface at top step edges, promoting bond cleavage for catalysis. DFT calculations confirm that the top step edge offers a stable, reactive adsorption environment, enhancing catalytic efficiency. By leveraging the coordination environment of step nickel atoms, we propose a model linking surface facets to catalytic behavior: (111) facets promote deep furan ring hydrogenation, (001) facets activate aldehyde groups, and step sites uniquely enable self-hydrogenation to 2-methylfuran. Understanding these distinct coordination environments is crucial for designing catalysts with tailored reactivity and selectivity, offering a targeted approach to enhance efficiency and selectivity in biomass conversion.

These insights lay a robust foundation for the design of advanced catalysts for the selective conversion of biomass. Moving forward, further exploration of spin density variations at atomic steps and the catalytic potential of pyramidically shaped nanoparticles may unlock additional pathways for enhancing selective hydrogenation and catalytic efficiency.

## Methods

The methods used in this study are described in detail in a previous publication^18^ and in the Supporting Information. However, a brief overview of the methods is provided here. We performed in-situ qualitative and quantitative chemical analysis of the nickel surface (5[001] × [111]) in an ultrahigh vacuum chamber, maintained at a base pressure of 5 × 10^–10^ mbar. The experimental setup included a quadrupole mass spectrometer (QMS-Balzers QMG 421), X-ray photoelectron spectroscopy (XPS Leybold LHS-12), ultraviolet photoelectron spectroscopy (UPS-Specs), low energy electron diffraction (Technology LEED-WA), an Ar^+^ ionization gun (Specs), and a vibrating Kelvin probe (VCKP-Delta Phi Elektronik). The nickel single crystal was resistively heated within a temperature range of 160–1250 K, with temperatures monitored by K-type thermocouples attached to both the cystal and its support. Prior to analysis, the sample underwent cleaning via repeated cycles of Ar^+^ bombardment and annealing at 1000 K. The surface clreanliness was verified using LEED, XPS, and UPS until no impurities were detected. Organic molecules, including furfural, furan, 2-methylfuran, and furfuryl alcohol (Sigma-Aldrich, 99%), as well as H_2_ (Energas, 99.99%) and CO (Energas, 99.999%), were purified using freeze–pump–thaw cycles before being introduced into the chamber at the required pressure for adsorption measurements.

UPS measurements utilized a HeI, HeII excitation source (21.22, 40.81 eV), negatively biased (12.28 V) to effectively separate the secondary electrons of the analyzer. Spectral analysis was conducted using the XPSpeak41, employing Gaussian peak shapes and Shirley background subtraction in all cases (details can be found in the Supporting Information). Measurement accuracy was approximately ±10 meV. Thermal desorption spectroscopy (TDS) and temperature-programmed reaction spectroscopy (TPRS) experiments were conducted with a heating rate of 1 K s^–1^. The mass spectrometry data were corrected for sensitivity and molecular ionization cross sections based on theoretical calculations (Nakao et al.^64^) and were consistent with the NIST database for furfural ionization.^65^ Desorbed species were identified through their molecular mass fragments. Semirelativistic density functional theory (DFT) calculations were performed using the ABINIT 10.0.3 package,^66−68^ employing slab models to estimate total energies and related structures, as described analytically in the Supporting Information.

## Supporting Information Available

Sections S1–S7, Figures S1–S16, and Tables S1–S6, which provide comprehensive experimental protocols and data analysis for TPD/TPR measurements, as well as surface characterization via XPS, UPS, and Kelvin probe (VCKP) measurements. Computational methods, including DFT calculations, address adsorption sites, bond deformations, and electronic states. Additionally, it describes methodologies for calculating RMSD, coordination numbers, and generalized coordination numbers, offering insights into structural and electronic modifications upon adsorption (PDF)
