# Supplementary material for: Mimicking Real Catalysts: Model Stepped Nickel Surfaces in Furfural Catalysis—Insights into Adsorption, Reactivity, and Defect-Driven Conversion Pathways
Source: J Phys Chem Lett. 2025 Mar 17;16(12):3022–33. doi: 10.1021/acs.jpclett.5c00066 (PMC11956134; doi:10.1021/acs.jpclett.5c00066)
Supplement: Supplementary file 1 — jz5c00066_si_001.pdf [file jz5c00066_si_001.pdf]

Supporting Information

Mimicking Real Catalysts: Model Stepped Nickel Surfaces in Furfural Catalysis—Insights into Adsorption, Reactivity, and Defect-Driven Conversion Pathways

Sotirios Tsatsos and Georgios Kyriakou*

Department of Chemical Engineering, University of Patras, Caratheodory 1, Patras GR 26504, Greece

E-mail: kyriakg@upatras.gr

Phone: +30 2610997287

Supporting Section 1. Thermal Desorption Spectra Quantification

Using the H_2 desorption peak, we can estimate the residual carbon content on the Ni(119) surface, in accordance with prior studies,[1,2] as depicted below.

$$C = \frac{P_{H_2} \cdot \sigma_{H_2}}{R_{H/C}} \tag{S1}$$

where, the variable denoted as P_{H_2} represents the peak area associated with H_2 desorption. The parameter σ_{H_2} denotes the sensitivity coefficient characterizing the response of molecular H_2 to the quadrupole mass spectrometer detection apparatus, which incorporates the ionization cross-section specific to H_2. Lastly, the parameter $R_{H/C}$ represents the molar ratio between hydrogen (H) and carbon (C) atoms in furfural ($C_5H_4O_2$). The determination of reaction selectivity for all experimental series is performed using the following expression:

$$S_i = \frac{P_i \cdot \sigma_i}{\sum_{i=1}^{n} (P_i \cdot \sigma_i)} \tag{S2}$$

where P_i represents the area of the i-th desorption peak, σ_i is the sensitivity coefficient, and n is the total number of desorbed peaks, including residual carbon contributions, as determined through the Eq. S1.

Supporting Section 2. Temperature Desorption Spectroscopy Data

The behavior of hydrogen on the Ni(119) surface as observed in the thermal desorption spectra, Figure **S1**, reflects the complex interplay between surface structure, hydrogen (H_a) coverage, and adsorption site occupation. The recombinative desorption can explain the

features observed in thermal desorption spectra of hydrogen on a Ni surface. The shift of the desorption peaks to lower temperatures with increasing hydrogen coverage is a direct consequence of the ease of recombination as H_a atoms occupy less strongly bound sites at higher coverages. The appearance of multiple peaks at higher coverages reflects the presence of different adsorption sites and recombination pathways on the Ni(119) surface.

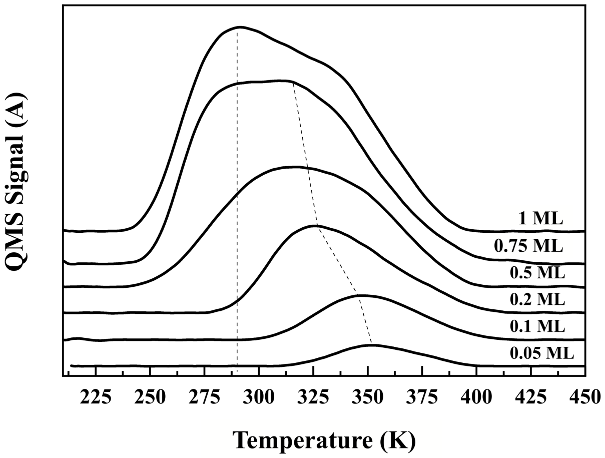

Figure S1: Thermal desorption spectra of H_2 on the clean Ni(119) surface at various coverages, ranging from 0.05 ML to 1 ML. The spectra were recorded with heating rate of of 1 Ks^{-1}, and adsorption was performed at approximately 230 K.

The desorption behavior of furfural on both pristine and carbon-passivated Ni(119) surfaces was investigated to elucidate its different reactivity pathways. The molecular mass fractions used for identifying the desorbed species were as follows: furfural (m/z: 18, 28, 39, 42, 53, 68, 96, 98), tetrahydrofuran (m/z: 28, 32, 39, 42, 72), furan (m/z: 18, 28, 32, 39, 42, 68), 2-methylfuran (m/z: 18, 28, 32, 39, 42, 53, 82), furfuryl alcohol (m/z: 39, 42, 53, 55, 68, 82, 96, 98), propene (m/z: 28, 39, 41), H_2 (m/z: 2), CO (m/z: 28), and H_2O (m/z: 18).

Figure **S2** displays temperature-programmed desorption (TPD) spectra of furfural on Ni(119) across coverages ranging from 0.25 to 2 monolayers (ML). Two desorption peaks at 200 K and 220 K indicate physically adsorbed and chemisorbed furfural, respectively. The main desorption products, 2-methylfuran (2MF) and furan, appear at 290 K and 270 K, respectively. The formation of 2MF is attributed to the initial hydrogenation followed by

hydrodeoxygenation of furfural, whereas furan results from the decarbonylation of furfural. Carbon monoxide desorption at 390 K, associated with furfural decarbonylation, aligns with chemisorbed CO on Ni(119). Therefore the evolution of CO after Furfural decarbonylation is a desorption-rate-limited process.[1] Hydrogen desorption, from the dehydrogenation and decomposition of furfural, occurs at 370 K and 470 K, with a 290 K peak represents the recombinative desorption of molecular H_2. Finally, mass 39 is crucial for identifying all reaction products, as it corresponds to a common fragment that results from the superposition of all hydrocarbon species detected during the TPD ramp, as shown in Figure **S2**(d).

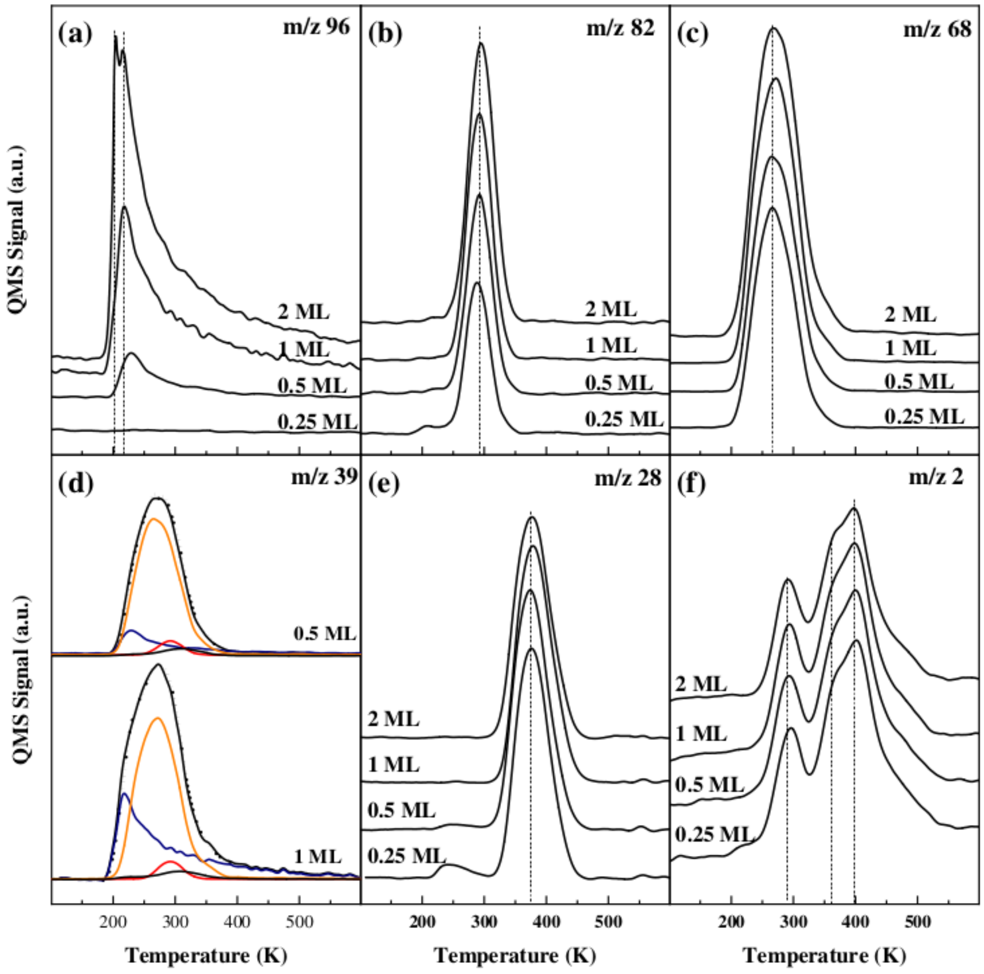

Figure S2: Desorption spectra of furfural (FUR) on the Ni(119) surface for coverages ranging from 0.25 to 2 ML. (a) FUR (m/z 96), (b) 2MF (m/z 82), (c) Furan (m/z 68), (d) Superposition m/z 39 (orange: furan, blue: FUR, red: 2MF), (e) CO (m/z 28), (f) H_2 (m/z 2). The spectra were recorded with heating rate of of 1 Ks^{-1}, after adsorption at 170 K.

Figure **S3** expands the study of furfural interactions on Ni(119) surfaces in the presence of residual carbon, a byproduct of the initial reaction cycle on the clean surface (see Figure **S11**). Furfural coverages range from 0.5 to 2 ML. As on the pristine surface, the carbon-passivated surface exhibits dual desorption peaks at 200 and 220 K. However, a notable shift in product distribution occurs: the carbon-modified surface predominantly yields furan (m/z 68) at 270 K, while hydrogenation and hydrodeoxygenation byproducts like 2MF are absent. This finding suggests that residual carbon directs selectivity toward furfural decarbonylation.

As discussed in the main paper the latter elucidates the direct influence of individual steps on the self-hydrogenation process, particularly through the spontaneous activation of C−H bonds. Structural changes in the catalytic surface are known to significantly affect reaction rates and product distributions, potentially leading to catalyst poisoning.[3] Previous work by Blakely, Isett, and Lang[4–6] showed that carbon deposition predominantly occurs along monatomic steps on vicinal (100) surfaces, achieving full monatomic coverage without structural reconstruction at higher carbon concentrations. Blakely and Somorjai[3] further demonstrated that carbon and oxygen form distinct bonds at various surface sites on platinum, resulting in structural changes. These findings indicate that carbon adsorption mainly occurs in surface regions with low charge density or electrophilic properties. Recent research has confirmed that these regions are primarily along monatomic steps due to the Smoluchowski effect.[7] Carbon deposition along these steps initially inhibits C−H bond activation, preventing the release of hydrogen necessary for furfural self-hydrogenation and leading to the exclusive formation of decarbonylation species. This conclusion is supported by the limited recombinative desorption of H_2 from the passivated surface compared to the clean surface, as shown in Figure **S3**(f). The presence of carbon on the Ni(119) surface impacts both selectivity and overall furfural conversion.

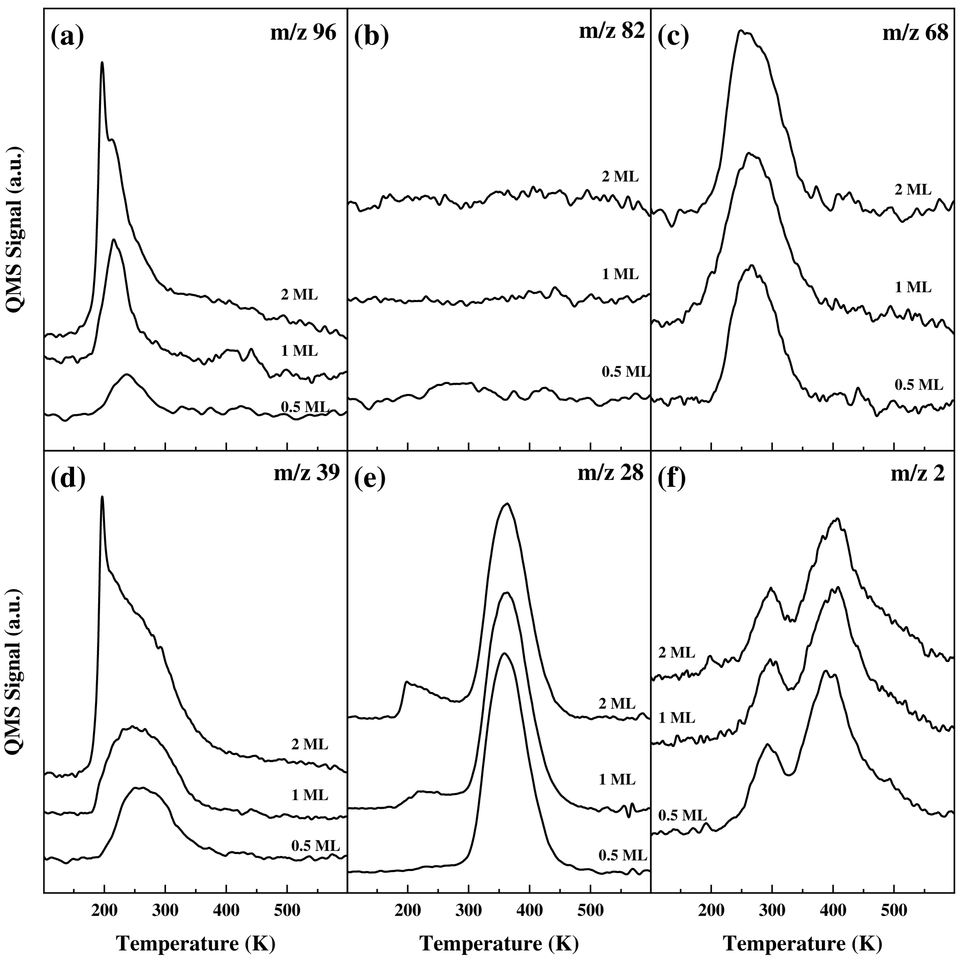

Figure S3: Desorption spectra of furfural (FUR) on the Ni(119) surface in the presence of residual carbon for coverages ranging from 0.5 to 2 ML. (a) FUR (m/z 96), (b) 2MF (m/z 82), (c) Furan (m/z 68), (d) m/z 39, (e) CO (m/z 28), (f) H_2 (m/z 2). The spectra were recorded with heating rate of of 1 Ks^{-1}, after adsorption at 170 K.

Figure **S4** and **S5** illustrate the desorption behavior of furfural at varying coverages in the presence of pre-adsorbed Hydrogen. In these experiments, a fraction of H_a (1-x) was first adsorbed, followed by the adsorption of furfural (x), ensuring the total coverage reached a monolayer. This approach enables a detailed analysis of the interaction dynamics between furfural and pre-adsorbed hydrogen across different coverage conditions. The desorption temperature of furfural (m/z 96) on the pristine Ni(119) surface remains constant at 220 K in the presence of H_2. Similarly, 2-methylfuran (2MF) consistently desorbs at 285 K, regardless of pre-adsorbed H_2, indicating a stable desorption profile. Notably, the desorption

behavior of furan, formed through surface reactions, exhibits significant variation. On the pristine Ni(119) surface, furan desorbs as a single peak at 270 K (Figure **S7**(a)). However, when furfural is co-adsorbed with H_2, the furan desorption spectrum shows three distinct peaks at 270, 300, and 350 K, highlighting a notable shift in behavior.

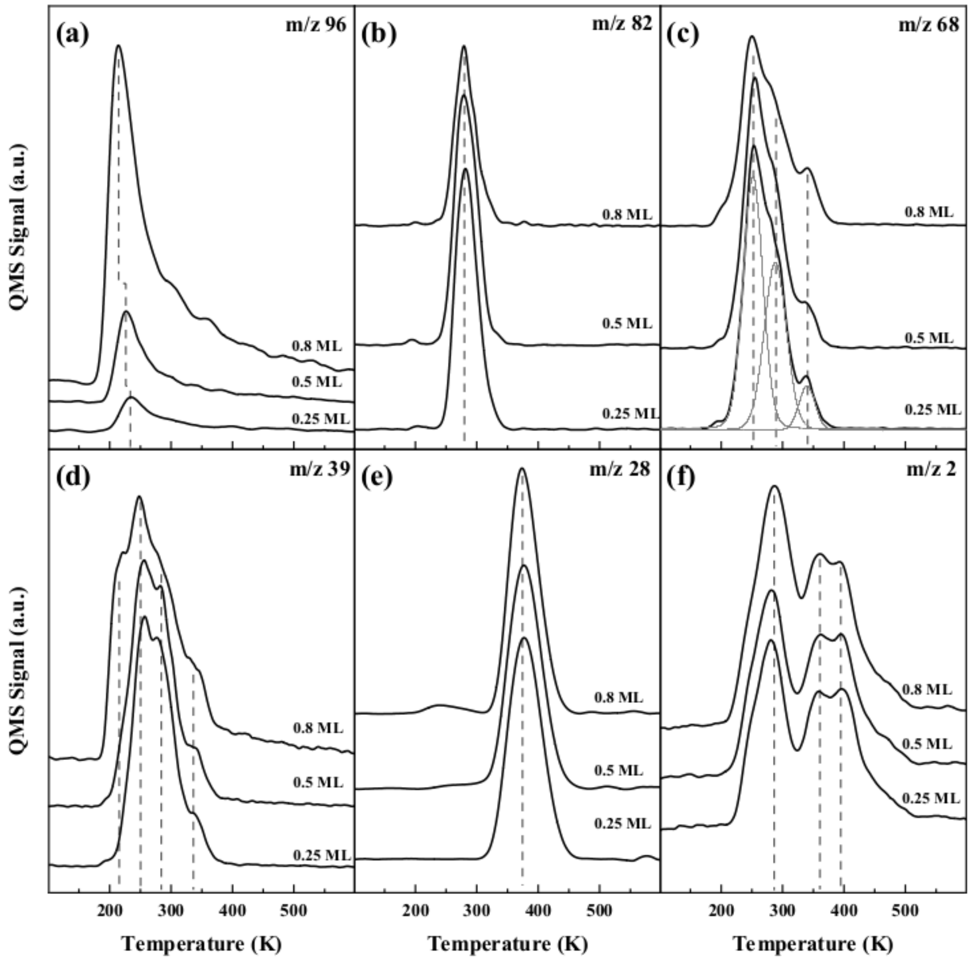

Figure S4: TPSR spectra of FUR (x) on the Ni(119) surface with varying pre-adsorbed H_2 amounts (1-x) at fractional coverages (x) of 0.25, 0.5, and 0.8 ML. (a) FUR (m/z 96), (b) 2MF (m/z 82), (c) Furan (m/z 68), (d) m/z 39, (e) CO (m/z 28), (f) H_2 (m/z 2).The spectra were recorded at a heating rate of 1 Ks^{-1}, after adsorption at 170 K.

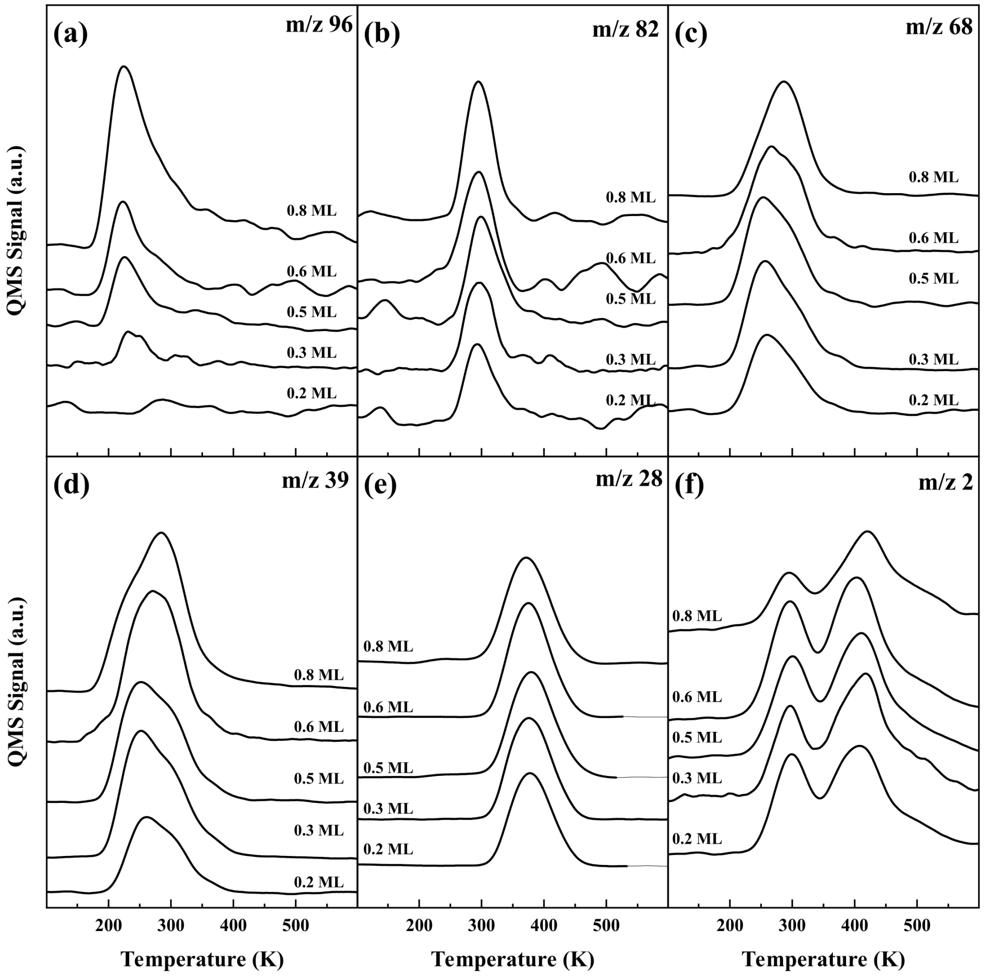

Figure S5: TPSR Spectra of FUR (x) on a passivated Ni(119) surface with pre-adsorbed H_2 (1-x) for coverages of $x = 0.2$ to 0.8 ML.(a) FUR (m/z 96), (b) 2MF (m/z 82), (c) Furan (m/z 68), (d) Fragment with m/z 39, (e) CO (m/z 28), (f) H_2 (m/z 2). The spectra were recorded with heating rate of of 1 Ks^{-1}, after adsorption at 170 K.

Figures **S6** and **S7** compare the TPD and TPSR spectra of furfuryl alcohol (FAL) and furan on a clean Ni(119) surface, both with and without pre-adsorbed H_a. In Figure **S6**, the furfural (Fur) coverage is 0.8 ML, while hydrogen coverage is 0.2 ML. For furan (Figure **S7**), both furan and hydrogen have coverages of 0.5 ML. These spectra illustrate the influence of hydrogen on desorption profiles and reaction pathways.

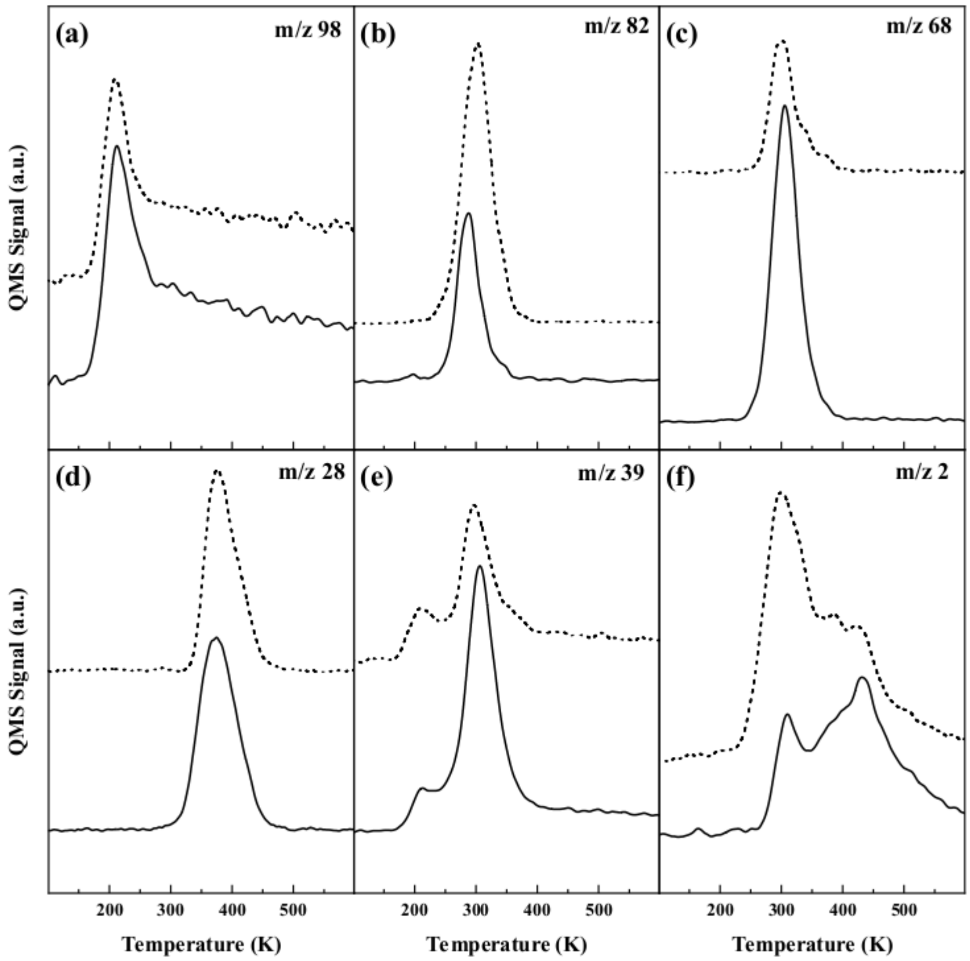

Figure S6: TPD and TPSR spectra of FAL on a clean Ni(119) in the absence (solid line) and presence of pre-adsorbed H_2 (dashed line). FAL coverage is 0.8 ML, while H_2 coverage is 0.2 ML. The spectra were recorded at a heating rate of 1 Ks^{-1}, after adsorption at 170 K.

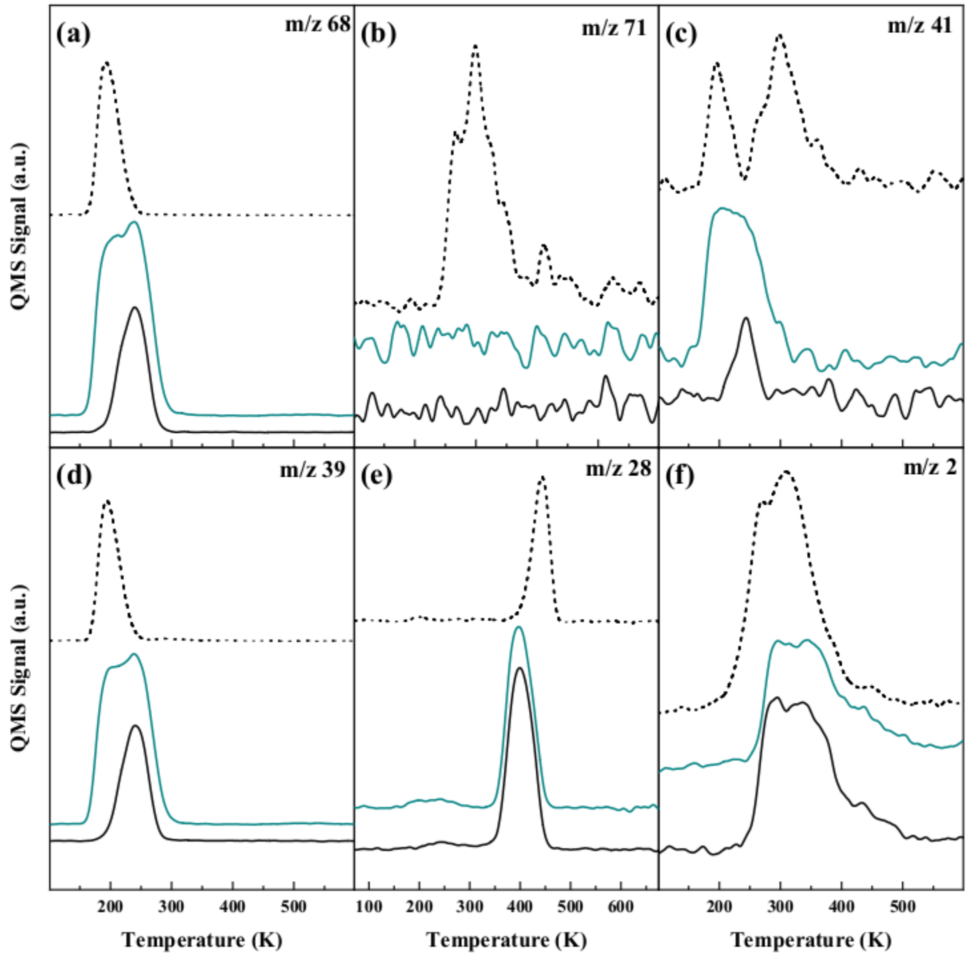

Figure S7: TPD and TPSR spectra of furan on a clean Ni(119) surface in the absence (solid line) and presence of pre-adsorbed H_2 (dashed line). Furan coverage is 0.5 ML, while H_2 coverage is 0.5 ML. The cyan solid line corresponds to 2 ML of furan. The spectra were recorded with heating rate of of 1 Ks^{-1}, and adsorption was performed at 170 K.

The desorption peak of furan during the desorption of 0.8 ML of furfuryl alcohol (FAL) on both the clean Ni(119) surface and in the presence of 0.2 ML H_a (Figure **S6**(c))is centered at 300 K, consistent with the intermediate desorption peak observed in the case of furfural desorption. This suggests a direct correlation of this peak with reactively formed FAL on the Ni(119) surface. Furthermore, the desorption of reactively formed CO due to decarbonylation and H_2 exhibits typical desorption characteristics of chemisorbed CO and H_2 on the Ni(119) surface (see Figure **S1**).[1] Clearly, the presence of pre-adsorbed H_a enhances the low-temperature desorption peak (recombinative H_2 desorption) compared to the

clean surface, indicating that only a portion of the total surface hydrogen participates in the hydrogenation and subsequent hydrodeoxygenation (HDO) of furfural to 2MF, while the remaining hydrogen desorbs without undergoing reaction.

To conclude, the transformation of FUR and FAL on Ni(119) surface follows distinct mechanistic pathways due to differences in their adsorption strength, reaction energetics, and desorption behaviors. Specifically, FAL displays a stronger interaction with the Ni(119) surface than FUR, which implies that FAL is more stable and remains adsorbed longer, necessitating additional energy for further reaction steps. The stronger adsorption of FAL results in a higher desorption temperature for furan, as FAL undergoes multiple steps before furan is formed and subsequently desorbed. In particular, FAL must first undergo hydrogenolysis to form FUR, followed by a decarbonylation step—an energy-intensive process involving C=O bond cleavage—to release furan.[8] In contrast, FUR can proceed directly to decarbonylation and form furan without requiring additional intermediate transformations. Consequently, furan desorbs from FAL at a higher temperature (300 K) compared to FUR (270 K), indicating a stepwise and gradual decomposition of FAL on the Ni(119) surface.

In the presence of H_a, the selectivity toward 2MF over furan is enhanced by facilitating critical hydrogenation steps for FAL conversion. Hydrogen is crucial for the HDO process that sequentially converts FUR to FAL and finally to 2MF.[9] When H_a is present, it reduces the activation barriers for key hydrogenation steps, enabling more efficient transformation of intermediates into fully hydrogenated products like 2MF. A hydrogen-rich Ni(119) surface favors pathways toward 2MF formation by preventing the accumulation of surface intermediates on the surface, which would otherwise occupy active sites and inhibit further hydrogenation reactions. In the absence of H_2, reactions reliant on hydrogenation tend to stall as surface hydrogen atoms are depleted, leading to higher surface coverage by intermediates and favors the desorption pathways over conversion to 2MF. Thus, H_2 plays a critical role in maintaining the catalytic cycle, by shifting reaction equilibria towards 2MF formation, thereby enhancing selectivity and conversion efficiency in the hydrogenation pathway.

Supporting Section 3. Surface Analysis

The C1s spectrum (Figure **S8**(b)) features four primary peaks: a graphitic sp^2 carbon peak at 284.1 eV, a $\pi-\pi^*$ shake-up transition peak around 291 eV, aliphatic and aromatic carbon bonds (C−C and C−H) at 285 eV, and peaks for functional groups of alcohols and esters (C−O−C and/or C−OH) around 286.5 eV, with a carbonyl bond peak at 287.7 eV.[10,11] The deconvolution details for C1s peaks are presented in Table **S1**. Table **S2** outlines the O1s region's deconvolution parameters, highlighting two primary carbon-oxygen bonds in aromatic compounds: the aromatic oxygen C−O−C at approximately 534.5 eV and the carbonyl group (C−C=O) peak at 532.2 eV, with a characteristic shake-up transition peak around 536 eV indicative of $\pi-\pi^*$ valence excitations accompanying core-shell ionization.[10]

The average furfural thickness was determined using Eq. S3. The inelastic mean free path (IMFP, denoted as λ) was calculated using the TPP-2M method, as described by Tanuma, Powell, and Penn,[12,13] which provided the following IMFP values: 1.10 nm for $\lambda_{Ni2p-Ni}$, 2.09 nm for $\lambda_{Ni2p-C_5H_4O_2}$, and 3.48 nm for $\lambda_{C1s-C_5H_4O_2}$, while the ratio $\frac{I_C^\infty}{I_{Ni}^\infty}$ was found to be 0.03.

$$\frac{I_{C1s}}{I_{Ni2p}} = \frac{I_{C1s}^\infty}{I_{Ni2p}^\infty} \cdot \frac{1 - e^{-\frac{d}{\lambda_{C1s-C_5H_4O_2}}}}{e^{-\frac{d}{\lambda_{Ni2p-C_5H_4O_2}}}} = 0.03\left(e^{0.47\cdot d} - e^{0.19\cdot d}\right) \tag{S3}$$

Table S1: Data on binding energies and spectral profiles for the fitting of the C1s region.

**Species**	**Binding Energy (eV)**	***Lineshape**	**FWHM (eV)**
**C**	284.1	TS G-L(30)	1.7
**C**−**C**, C−H (C_1−C_2)	285.1	TS G-L(30)	1.7
**C**−O−**C** (C_3−C_4)	286.3	G-L(30)	1.7
**C**=O, **C**−**C**=O (C_5)	287.6	G-L(30)	1.7
O−**C**=O	289.0	G-L(30)	1.7
$\pi-\pi^*$	292.0	G-L(30)	3.0

** TS denotes an asymmetric tail, while G-L represents a Gaussian-Lorentzian lineshape.*

Table S2: Data on binding energies and spectral profiles for the fitting of the O1s region.

Species	Binding Energy (eV)	Lineshape	FWHM (eV)
C−**O**−C (O_1)	534.6	G-L(30)	2
C−C=**O** (O_2)	532	G-L(30)	2
C−C=**O** (shake-up)	536	G-L(30)	2

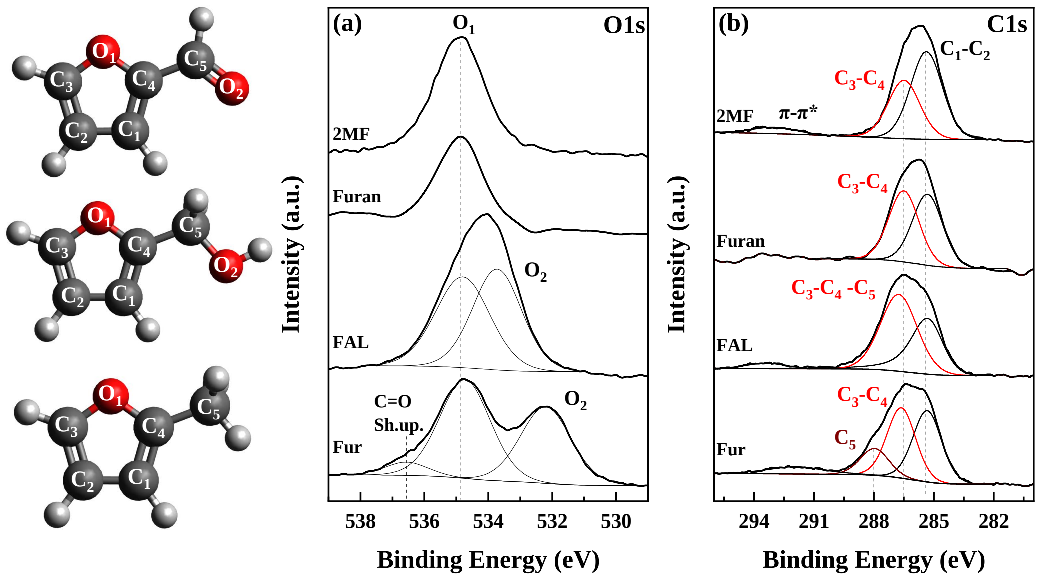

Figure S8: XPS reference spectra for the C1s and O1s regions of furfural, furan, 2MF, and FAL molecules, each at 1 ML coverage, were obtained at 170 K.

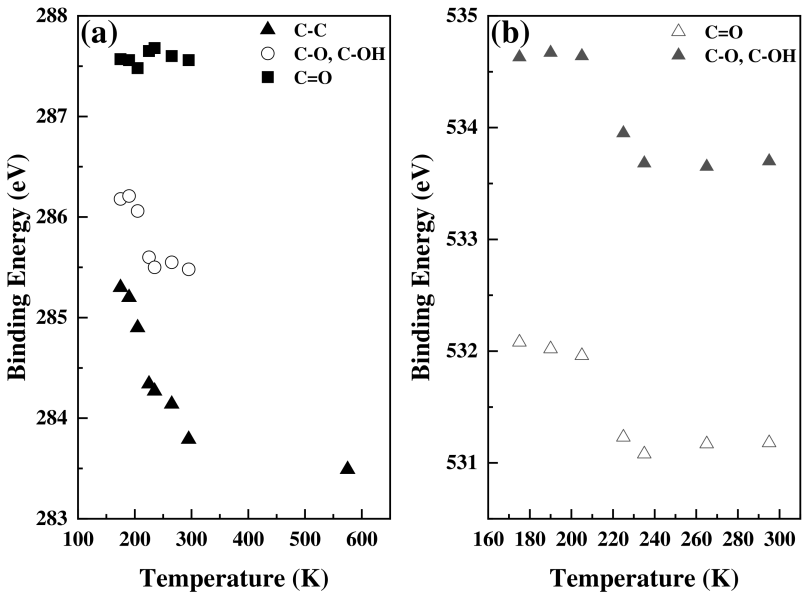

Figure S9: Variation in the binding energies of various carbon (a) and oxygen (b) species as a function of annealing temperature. The peak assignments are denoted using a general notation to account for different chemical species that may form during furfural desorption in the annealing process. For instance, the C−C bond is represented by C_1-C_2, corresponding to specific carbon species.

Table S3: Data showing the variation of Ni, C, and O content, O/C ratio, film thickness, and carbon reduction percentage as a function of temperature.

Temperature (K)	Ni	C	O	O/C	Thickness (nm)	Carbon Reduction (%)
175	1.00	1.04	0.43	0.41	1.69	0.00%
190	1.00	0.46	0.19	0.41	0.95	55.77%
205	1.00	0.22	0.09	0.41	0.52	78.85%
225	1.00	0.09	0.04	0.44	0.23	91.35%
235	1.00	0.08	0.03	0.41	0.20	92.31%
265	1.00	0.06	0.02	0.36	0.14	94.71%
295	1.00	0.05	0.02	0.30	0.13	95.19%
575	1.00	0.04	0.00	0.00	0.04	96.15%

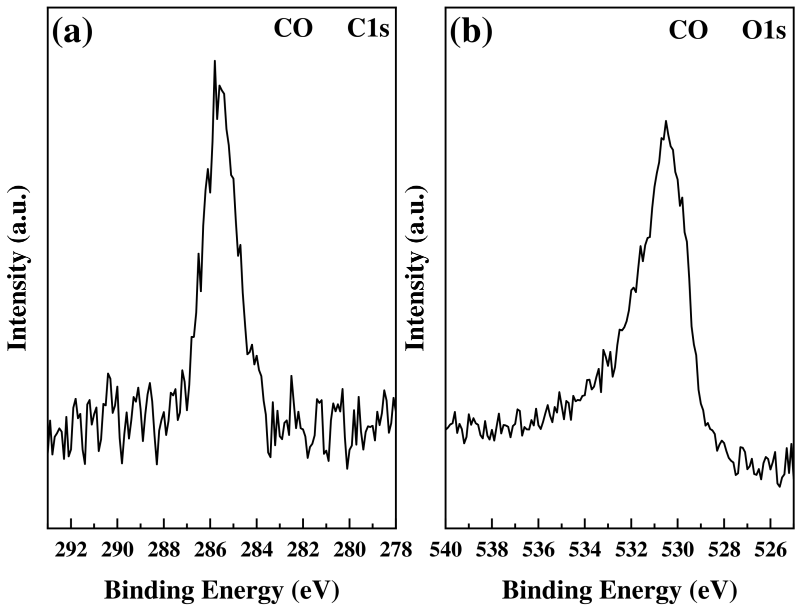

Figure S10: XPS spectra for the C1s and O1s regions after saturating the Ni(119) surface with CO. It was observed that CO predominantly adsorbs at the bridge sites of the nickel surface since the peak of the C1s spectrum is observed at a binding energy of 285.6 eV. In parallel, the peak corresponding to oxygen, within the O1s spectrum, is identified at a binding energy of 531 eV.[1,14,15]

Figure **S11** shows carbon deposition on a Ni(119) surface during furfural desorption cycles. Subplot (a) indicates a linear increase in carbon coverage, with each cycle adding approximately 0.12 monolayers. Subplot (b) demonstrates that carbidic carbon forms consistently during the first three cycles, saturating the surface, while graphitic carbon starts forming at 550 K after these cycles. This suggests that the stepped Ni(119) surface facilitates carbide formation at lower temperatures and promotes the transition to graphite, due to its enhanced reactivity and adsorption properties. The observed carbides are likely localized on the surface rather than forming a bulk phase. Notably, carbon deposition on Ni based catalysts during furfural hydrogenation in continuous-flow gas-phase reactors has been shown to result in the formation of different carbon species including polymeric structures, which may influence reaction activity and selectivity.[16] Future studies using Raman spectroscopy on supported catalysts in atmospheric conditions could offer valuable insights into carbon diffusion, buildup, and transformation at a larger scale, complementing the atomic-level insights

obtained from single-crystal studies.[17,18]

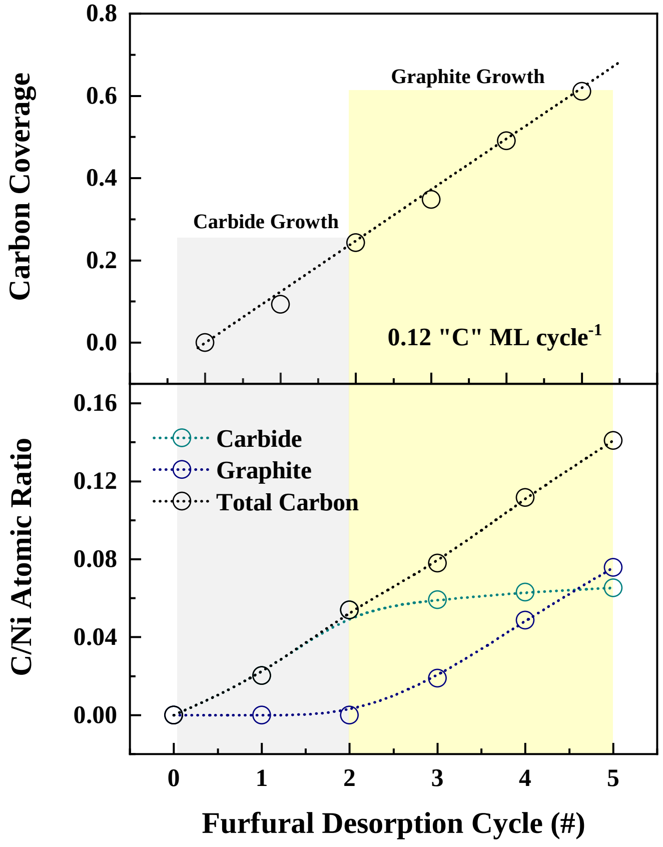

Figure S11: Quantitative analysis of carbon deposition on Ni(119) surface.(a) Carbidic and graphitic carbon coverage in monolayers, based on the orthogonal epitaxy lattice parameter (0.246 Å). (b) Atomic ratio of C1s to Ni2p, following sequential furfural exposures at 180 K and annealing at 550 K.

The ionization energies (IEs) in Figure **S12** were calculated using DFT with the B3LYP functional and an augmented basis set, as previously detailed[19] using the GAMESS (US) package.[20,21] The IEs were adjusted for the work function of Ni(119) to align with experimental conditions. The observed binding energy shifts in the UPS spectra, relative to the calculated IEs, are described by the expression $E_{ads} = E_{gas} - (\phi + \Delta\phi) + (E_{relax} + E_{bond\ shift})$. Here, E_{ads} and E_{gas} are the IEs for the adsorbed and gas phases, respectively. E_{relax} accounts for final state relaxation, while $E_{bond\ shift}$ represents the initial state shift due to adsorbate-substrate interactions. The terms ϕ and $\Delta\phi$ account for changes in the work function. These energy shifts arise from the strong coupling between the adsorbate and the Ni(119) substrate.

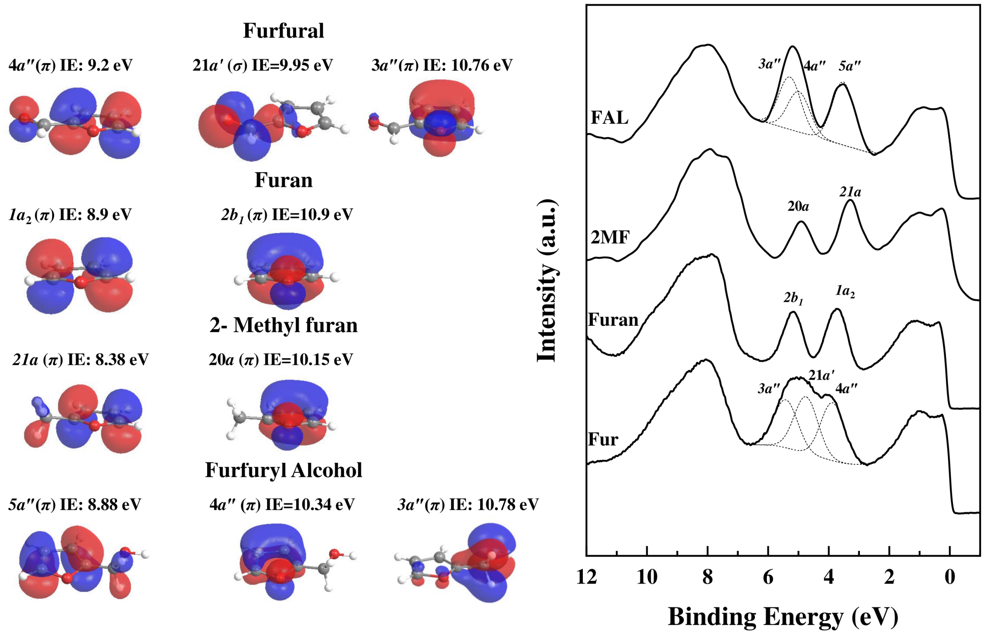

Figure S12: UPS HeI emission spectra of furfural (Fur) and derivatives furan (Furan), 2-methylfuran (2MF), and furfuryl alcohol (FAL) following ~0.6 ML absorption at 175 K, with accompanying highest occupied molecular orbitals (HOMOs) and gas-phase ionization energies (IEs). The ionization energy obtained through UPS measurements exhibits a shift relative to gas-phase spectra. This deviation is attributed to the significant interaction between the adsorbed molecule and the substrate, as described in detail in a previous study.[19]

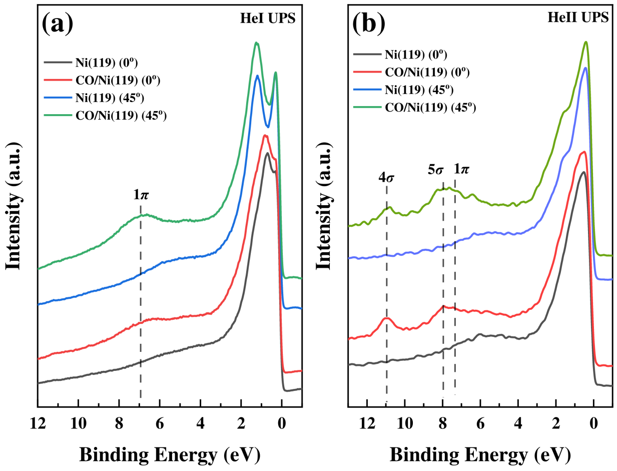

Figure S13: Comparative UPS spectra of CO adsorbed on Ni(119) surface using (a) HeI photon energy (21.2 eV) and (b) HeII photon energy (40.8 eV). The analysis highlights the polar-dependent cross-section of the CO molecule 4σ and 1π MOs, with the polarization vector aligned parallel and perpendicular to the molecular axis, respectively. The data illustrate the significant variation in energy cross-section between the two photon energies, textitasizing the diminished visibility of spectral peaks at 21.2 eV compared to 40.8 eV.[22,23]

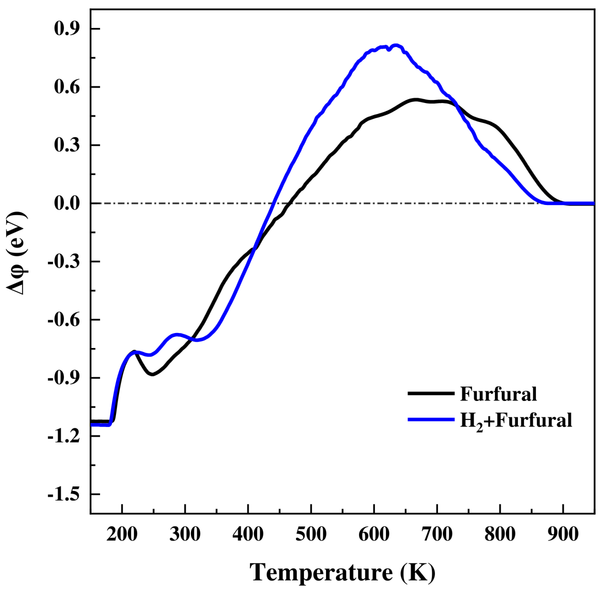

Figure S14: Alteration in the work function ($\Delta\phi$) throughout the thermal desorption of furfural (1 ML), a combination of furfural (0.8 ML) with hydrogen (0.2 ML) on Ni(119) surface, with a heating rate of 1 Ks^{-1}.

Supporting Section 4. Computational Details

All calculations were performed using the ABINIT 10.0.3 package[24–26] within the framework of spin-unpolarized Density Functional Theory (DFT). The Generalized Gradient Approximation (GGA) with the Perdew-Burke-Ernzerhof (PBE) functional was employed to describe the exchange-correlation interactions.[27] A plane wave basis set with a cutoff energy of 700 eV and optimized norm-conserving Vanderbilt pseudopotentials were utilized.[28] For bulk Ni calculations, the Brillouin zone was sampled using a Monkhorst-Pack grid[28] of $8 \times 8 \times 8$. The occupation scheme was managed using Methfessel-Paxton smearing method with a width of 0.1 eV.[29] The optimized bulk lattice parameters were determined to be $a = b = c = 3.525 Å$, which closely match the experimental value of 3.524 Å. For the stepped Ni(119) surface, a three-layer slab model with a 4×1 supercell was constructed. A 15 Åvacuum slab was added to minimize interactions between periodic images. During the optimization process, the lowest atomic layer, consisting of sixteen atoms, was fixed, while the remaining two layers

were allowed to relax. Surface calculations were performed using a $2\times2\times1$ Monkhorst-Pack grid with a **k**-point shift of 0.5, 0.5, 0.5. Geometry optimizations required forces below 0.01 eVÅ^{-1} and energy changes below 5.0×10^{-5} eV/atom. SCF convergence was enhanced using a second-order damping algorithm with up to 8 Pulay iterations. Preconditioning involved calculating the RPA dielectric matrix at initial and later steps to manage the electronic response and improve SCF convergence.

In summary, the methods and parameters were chosen to ensure accuracy and stability in modeling the Ni(119) surface and furfural adsorption. Methfessel-Paxton smearing was used for its convergence properties in metallic systems, norm-conserving pseudopotentials for their balance of efficiency and accuracy, and an advanced preconditioning scheme for handling complex electronic structures effectively. This setup enables reliable simulations and insights into surface interactions and furfural adsorption on nickel.

Supporting Section 5. Computational Results and Discussion

The Figure **S15** illustrates the different adsorption sites of furfural on a stepped nickel surface, categorized into three distinct regions: Top Step Edge (S), Terrace (T), and Underneath Step (U). Each category comprises various configurations (S_2, S_3, S_4; T_2, T_3, T_4; U_2, U_3) representing different adsorption geometries and bonding interactions. In the Top Step Edge (S) region, the configurations (S_2, S_3, S_4) show significant interactions with the nickel atoms at the step edge. Configuration S_2 involves η^5-$\pi(C_1C_4)$-di$\sigma(C_2C_3)$-$\sigma(O_2)$ coordination, indicating a strong interaction with an adsorption energy of -1.79 eV. Configuration S_3, characterized by η^6-di$\sigma(C_1C_4)$-$\pi(C_2C_3)$-di$\sigma(C_5O_2)$, shows a slightly lower adsorption energy of -1.77 eV. Configuration S_4 exhibits η^3-$\sigma(C_1)$-di$\sigma(C_5O_2)$ coordination, resulting in an adsorption energy of -1.44 eV. These configurations at the top step edge demonstrate substantial adsorption energies, indicating strong bonding interactions between furfural and

the nickel atoms located at the step edge.

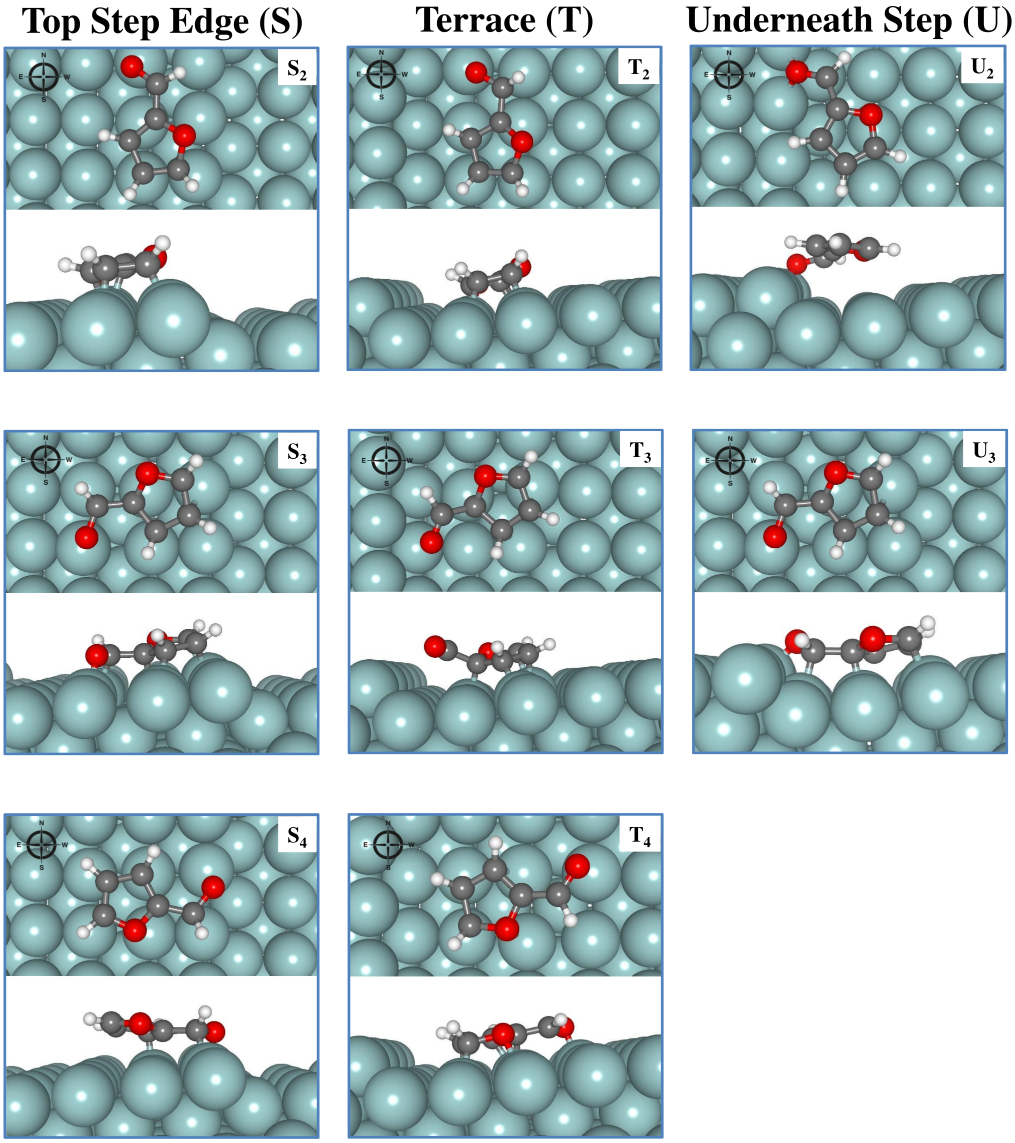

Figure S15: Adsorption sites of furfural on a stepped nickel surface categorized into three regions: Top Step Edge (S), Terrace (T), and Underneath Step (U). The configurations within each region (S_2, S_3, S_4; T_2, T_3, T_4; U_2, U_3) illustrate varying adsorption geometries and interactions. Adsorption strength decreases from the Top Step Edge (S) with the highest interaction energies, through the Terrace (T), to the weakest interactions underneath the step (U), highlighting the importance of step-edge sites in catalytic processes involving furfural.

In the Terrace (T) region, configurations (T_2, T_3, T_4) generally show moderate adsorption energies, indicating relatively less interaction strength compared to the top step edge configurations. Configuration T_2 features η^6-diσ(C_1C_4)-diσ(C_2C_3)-diσ(C_5O_2) coordination

with an adsorption energy of -1.63 eV. Configuration T_3 involves η^4-diσ(C_1C_4)-π(C_2C_3), resulting in an adsorption energy of -1.12 eV. Configuration T_4 exhibits η^6-π(C_1C_4)-diσ(C_2C_3)-σ(O_1)-σ(O_2) coordination with an adsorption energy of -1.45 eV. In the underneath step (U) region, configurations (U_2, U_3) show the lowest adsorption energies among all sites, reflecting weaker interactions and less favorable adsorption geometries. Configuration U_2 involves η^1-σ(O_2-NiNi) coordination with a lower adsorption energy of -0.87 eV. Configuration U_3 is characterized by η^6-π(C_1C_4)-π(C_2C_3)-diσ(C_5O_2), resulting in an adsorption energy of -1.32 eV. The figure clearly depicts the adsorption characteristics of furfural on different regions of a stepped Ni surface. Adsorption is strongest at the top step edge (S), followed by the terrace (T), and is weakest underneath the step (U). This hierarchy of adsorption strength is crucial for understanding surface chemistry and the catalytic properties of nickel surfaces in reactions involving furfural.

Table **S4** presents the results of the DFT calculation examining the adsorption of furfural on a stepped nickel surface. Various configurations and their corresponding adsorption energies, as well as RMSD values for both the nickel surface and furfural, are listed. The configurations are defined by the coordination of furfural with the Ni atoms, with the adsorption energy indicating the strength of interaction (more negative values denote stronger adsorption). The RMSD values provide insight into the structural deformation of both the nickel surface and the furfural molecule upon adsorption.

Table S4: Adsorption configurations, energies, and structural deformations of furfural on Ni(119) surfaces from DFT calculations. The table includes coordination types, adsorption energies (in eV), and root-mean-square deviation (RMSD) metrics for distinctive sites: S_1 (top step edge), T_1 (terrace), and U_1 (bottom step edge).

Configuration	Coordination	E_{ads} (eV)	RMSD (Å)	
			Ni Surface	Furfural
S_1	η^6-$\pi(C_1C_4)$-$\pi(C_2C_3)$-di$\sigma(C_5O_2$-NiNi)	-1.80	0.146	1.677
T_1	η^5-$\pi(C_1C_2)$-$\sigma(C_3)$-di$\sigma(C_5O_2$-NiNi)	-1.48	0.120	1.674
U_1	η^5-$\pi(C_1C_2)$-di$\sigma(C_3O_1)$-$\sigma(O_2)$	-0.82	0.110	1.674
S_2	η^5-$\pi(C_1C_4)$-di$\sigma(C_2C_3)$-$\sigma(O_2)$	-1.79	0.074	1.679
T_2	η^6-di$\sigma(C_1C_4)$-di$\sigma(C_2C_3)$-di$\sigma(C_5O_2)$	-1.63	0.060	1.665
U_2	η^1-$\sigma(O_2$-NiNi)	-0.87	0.110	1.675
S_3	η^6-di$\sigma(C_1C_4)$-$\pi(C_2C_3)$-di$\sigma(C_5O_2)$	-1.77	0.097	1.658
T_3	η^4-di$\sigma(C_1C_4)$-$\pi(C_2C_3)$	-1.12	0.116	1.661
U_3	η^6-$\pi(C_1C_4)$-$\pi(C_2C_3)$-di$\sigma(C_5O_2)$	-1.32	0.089	1.665
S_4	η^3-$\sigma(C_1)$-di$\sigma(C_5O_2)$	-1.44	0.071	1.670
T_4	η^6-$\pi(C_1C_4)$-di$\sigma(C_2C_3)$-$\sigma(O_1)$-$\sigma(O_2)$	-1.45	0.085	1.701

** The atom indices correspond to those shown in Figure **S8**.*

Table **S5** details the bond deformation of furfural upon adsorption on a stepped nickel surface, as derived from DFT calculations. Various configurations of furfural adsorption and the corresponding percentage changes in bond lengths for key bonds in the furfural molecule are presented. Positive values indicate bond elongation, while negative values indicate bond contraction. The results highlight how different adsorption configurations impact the structural integrity of furfural, revealing significant variations in bond deformation across different sites and bonding modes.

Table S5: Bonds deformation of Furfural upon adsorption on stepped nickel surface.

Configuration	C_1-C_2	C_2-C_3	C_3-O_1	O_1-C_4	C_4-C_1	C_4-C_5	C_5-O_2	C_1-H	C_2-H	C_3-H	C_5-H
S_1	1.8%	7.6%	6.3%	-1.9%	5.9%	-2.0%	13.5%	0.3%	0.4%	0.9%	-2.0%
T_1	-0.7%	0.5%	0.0%	0.1%	1.5%	-3.1%	6.2%	0.3%	0.0%	0.0%	-1.7%
U_1	3.2%	4.1%	3.4%	1.8%	4.5%	1.8%	9.7%	0.6%	0.4%	0.4%	-0.4%
S_2	3.3%	3.8%	3.6%	0.8%	8.2%	-1.8%	12.6%	1.6%	0.3%	0.1%	-2.3%
T_2	0.0%	0.0%	0.0%	0.0%	0.0%	0.0%	0.0%	0.0%	0.0%	0.0%	0.0%
U_2	3.3%	4.0%	1.9%	4.0%	9.9%	2.7%	0.0%	1.4%	0.3%	0.4%	-0.3%
S_3	3.6%	7.9%	7.1%	1.0%	4.4%	-1.3%	6.1%	0.3%	0.6%	1.4%	-1.3%
T_3	5.2%	5.0%	4.1%	2.8%	8.1%	2.2%	10.7%	1.3%	0.5%	0.4%	-1.2%
U_3	0.0%	0.0%	0.0%	0.0%	0.0%	0.0%	0.0%	0.0%	0.0%	0.0%	0.0%
S_4	4.8%	8.3%	6.5%	1.6%	7.3%	2.0%	10.4%	1.1%	1.1%	0.8%	-1.3%
T_4	0.2%	7.9%	9.4%	-0.1%	4.0%	-4.3%	7.3%	0.2%	0.2%	1.8%	-1.0%

** The atom indices correspond to those shown in Figure **S8**.*

Table **S6** presents the changes in Hirshfeld charges of furfural upon adsorption on a stepped nickel surface, as obtained from DFT calculations. The table reports the charge difference (Δe) for each atom across different adsorption configurations, along with the net charge redistribution for the furanic ring and the aldehyde group. For each configuration, the differential charge of the furanic ring is computed as:

$$\sum_{\text{ring}} q_i - 0.11e \tag{S4}$$

where $0.11e$ represents the initial charge of the furanic ring in the isolated furfural molecule. Similarly, the aldehyde differential charge is given by:

$$\sum_{\text{aldehyde}} q_i + 0.11e \tag{S5}$$

where $-0.11e$ is the initial charge of the aldehyde group in the isolated molecule, and rearranging leads to the addition of $0.11e$ in the formula. The total charge redistribution within the molecule is expressed as:

$$\sum_{\text{furfural}} q_i \tag{S6}$$

where summations extend over the respective atomic groups. Since the total charge of the isolated molecule is zero, this expression directly represents the net electron transfer upon adsorption. Negative values indicate electron accumulation, while positive values denote electron depletion. These results elucidate the electronic reorganization of furfural upon adsorption, highlighting the influence of different adsorption geometries on charge transfer with the Ni surface.

Table S6: Hirshfeld charge redistribution of Furfural upon adsorption on stepped nickel surface

Configuration	Δe (electrons)											Sum (electrons)		
	C_1	C_2	C_3	C_4	C_5	O_1	O_2	H_1	H_2	H_3	H_4	Furan	Aldehyde	Total
S_1	-0.08	-0.06	0.01	0.05	0.00	-0.08	-0.15	0.06	0.05	0.06	0.05	-0.10	0.01	-0.09
T_1	-0.07	-0.06	-0.01	0.07	-0.01	-0.07	-0.15	0.06	0.06	0.05	0.05	-0.08	0.00	-0.08
U_1	-0.07	-0.04	-0.01	0.05	0.02	-0.05	-0.18	0.05	0.05	0.06	0.03	-0.07	-0.02	-0.09
S_2	-0.05	-0.07	-0.01	0.04	0.05	-0.07	-0.19	0.06	0.06	0.06	0.04	-0.09	0.01	-0.08
T_2	-0.07	-0.07	-0.01	0.04	0.01	-0.08	-0.18	0.05	0.06	0.06	0.05	-0.13	-0.01	-0.14
U_2	-0.05	-0.06	0.04	0.04	0.06	-0.05	-0.13	0.05	0.06	0.06	0.05	-0.02	0.09	0.07
S_3	-0.08	-0.06	0.01	0.04	-0.01	-0.08	-0.18	0.05	0.06	0.06	0.05	-0.11	-0.03	-0.14
T_3	-0.07	-0.06	0.01	0.02	0.08	-0.08	-0.21	0.05	0.05	0.06	0.03	-0.13	0.01	-0.12
U_3	-0.04	-0.07	0.02	0.04	0.01	-0.07	-0.19	0.06	0.06	0.06	0.03	-0.05	-0.04	-0.09
S_4	-0.08	-0.06	0.03	0.07	-0.01	-0.04	-0.18	0.05	0.06	0.07	0.05	-0.01	-0.03	-0.04
T_4	-0.05	-0.07	-0.01	0.04	0.05	-0.05	-0.18	0.06	0.05	0.05	0.04	-0.09	0.02	-0.07
Isolated Furfural	-0.05	-0.07	0.03	0.04	0.09	-0.05	-0.24	0.07	0.07	0.07	0.04	0.11	-0.11	0.00

* *The atom indices correspond to those shown in Figure* ***S8***.

Figure **S16** shows the projected density of states (pDOS) for the termination layer of a Ni stepped surface in its clean state and following furfural adsorption in various configurations. The pDOS plots provide insights into changes in the electronic structure and the nature of the interaction between the furfural molecule and the Ni surface, forming the basis for calculating the differential pDOS.

The differential DOS, denoted $\Delta g(E)$, captures the impact of adsorption by measuring the change in electronic states, defined as:

$$\Delta g(E) = g_{\text{adsorbed}}(E) - g_{\text{clean}}(E) \tag{S7}$$

where $g_{\text{adsorbed}}(E)$ and $g_{\text{clean}}(E)$ represent the DOS for the adsorbed and clean surface configurations, respectively. To analyze these changes in detail, we integrate $\Delta g(E)$ over the energy ranges associated with occupied and unoccupied states relative to the Fermi level E_F:

$$\Delta n_{\text{occ}} = \int_{-10}^{E_F} \Delta g(E)\, dE \tag{S8}$$

$$\Delta n_{\text{unocc}} = \int_{E_F}^{10} \Delta g(E)\, dE \tag{S9}$$

The occupied states (Δn_{occ}) represent energies below the Fermi level (up to E_F), while unoccupied states (Δn_{unocc}) lie above E_F. These integrations provide a quantitative measure of how adsorption modifies the electronic states both below and above the Fermi level, offering insights into the redistribution of electronic density and potential changes in the adsorption and catalytic properties of the surface.

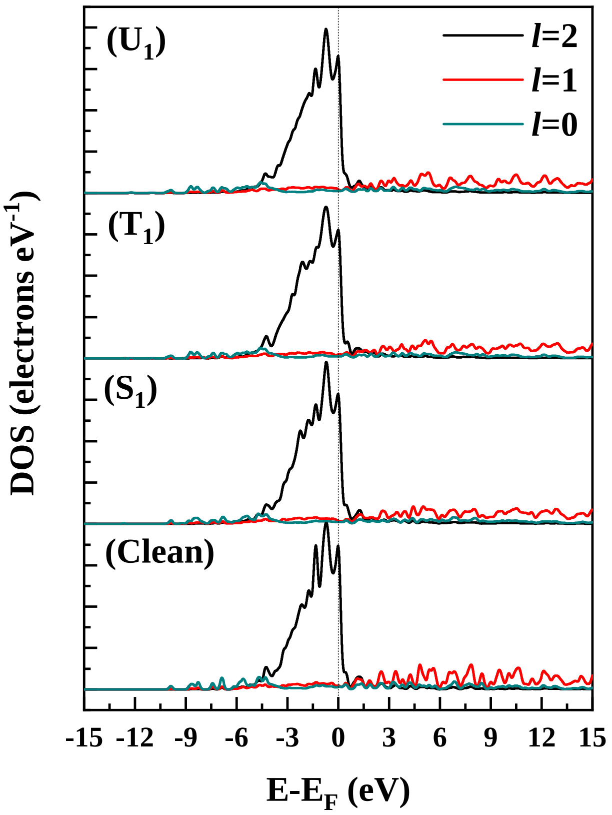

Figure S16: Projected density of states (pDOS) for the termination layer of a Ni stepped surface (Ni119) in its clean state and upon different furfural adsorption configurations.

Supporting Section 6. Analytical Description for RMSD Calculation Using the Kabsch Method

We calculate the Root Mean Square Deviation (RMSD) between the topmost atoms of pure and adsorbed Ni surfaces using the Kabsch algorithm.[30] First, the atomic coordinates are extracted from CIF files and converted from fractional to Cartesian using the lattice

parameters. The coordinates are centered by subtracting the centroid, $\mathbf{C} = \frac{1}{N}\sum_{i=1}^{N}\mathbf{r}_i$, where $\mathbf{r}_i$ are the atomic positions.

Next, we align the two structures by calculating the optimal rotation matrix, $\mathbf{R}$, via Singular Value Decomposition (SVD) of the covariance matrix $\mathbf{A} = \mathbf{X}_{\text{pure}}^T\mathbf{X}_{\text{adsorbed}}$, where $\mathbf{X}_{\text{pure}}$ and $\mathbf{X}_{\text{adsorbed}}$ are the centered coordinates. The matrix $\mathbf{A}$ is decomposed as $\mathbf{A} = \mathbf{U}\Sigma\mathbf{V}^T$, and the optimal rotation matrix is $\mathbf{R} = \mathbf{U}\mathbf{V}^T$.

Finally, the RMSD is calculated between the aligned sets of coordinates as:

$$\text{RMSD} = \sqrt{\frac{1}{N}\sum_{i=1}^{N}\left\|\mathbf{r}_{i,\text{pure}} - \mathbf{r}_{i,\text{adsorbed, rotated}}\right\|^2} \tag{S10}$$

This provides a quantitative measure of the structural difference between the two surfaces after alignment which is crucial for accurately quantifying the structural deviation between the pure and adsorbed surfaces. By aligning the structures optimally, we ensure that the measured difference reflects true atomic displacements, providing insights into how adsorption affects surface structure.

Supporting Section 7. Coordination Number and Generalized Coordination Number Calculation

The coordination number (cn) and generalized coordination number ($\overline{cn}$) were calculated for all atoms on the surface of the slab. The atomic positions were extracted from a CIF file and converted from fractional to Cartesian coordinates using lattice vectors derived from the lattice parameters. Pairwise distances $d_{ij} = \|\mathbf{r}_i - \mathbf{r}_j\|$ are computed between the selected atom i and all others, identifying neighbors within a cutoff distance. The coordination number (cn) for each atom is the count of neighbors within the cutoff:

$$cn_i = \sum_{j\neq i}[(d_{ij} \leq d_{\text{cutoff}}] \tag{S11}$$

The generalized coordination number ($\overline{cn}$) incorporates the coordination of neighbors, providing a more comprehensive measure. More specifically, the generalized coordination number $\overline{cn}$, for atom i is calculated by weighting the cn of each neighboring atom relative to the maximum coordination number observed among neighbors:

$$\overline{cn}_i = \sum_{j \in \text{neighbors of } i} \frac{cn_j}{\max_{k \in \text{neighbors of } i}(cn_k)} \tag{S12}$$

Matching cn and $\overline{cn}$ ensures that atoms in different structures have the same chemical and electronic environments, making them structurally equivalent. While cn reflects the number of nearest neighbors, $\overline{cn}$ accounts for the neighbors coordination, capturing the extended atomic network.

Generalized coordination numbers are particularly valuable as they incorporate both first and second nearest neighbors, offering a more descriptive measure than conventional coordination numbers. They correlate with adsorption energies and catalytic activity, especially in transition metals, and are linked analytically to the d-band center, a key descriptor for adsorption trends. This makes $\overline{cn}$ an effective and inexpensive descriptor for understanding adsorption energies and electrocatalytic performance across surfaces and nanoparticles.[31]

References

(1) Tsatsos, S.; Kyriakou, G. Copper Growth on a Stepped Nickel Surface: Electronic and Geometric Effects on CO Reactivity. *J. Phys. Chem. C* **2023**, *127*, 6337–6346, DOI: 10.1021/acs.jpcc.3c00377.

(2) Taylor, M. J.; Jiang, L.; Reichert, J.; Papageorgiou, A. C.; Beaumont, S. K.; Wilson, K.; Lee, A. F.; Barth, J. V.; Kyriakou, G. Catalytic Hydrogenation and Hydrodeoxygenation of Furfural over Pt(111): A Model System for the Rational Design and Operation of Practical Biomass Conversion Catalysts. *J. Phys. Chem. C* **2017**, *121*, 8490–8497, DOI: 10.1021/acs.jpcc.7b01744.

(3) Blakely, D. W.; Somorjai, G. A. The stability and structure of high miller index platinum crystal surfaces in vacuum and in the presence of adsorbed carbon and oxygen. *Surf. Sci.* **1977**, *65*, 419–442, DOI: 10.1016/0039-6028(77)90457-5.

(4) Eizenberg, M.; Blakely, J. M. Carbon interaction with nickel surfaces: Monolayer formation and structural stability. *J. Chem. Phys.* **1979**, *71*, 3467–3477, DOI: 10.1063/1.438736.

(5) Isett, L. C.; Blakely, J. M. Binding energies of carbon to Ni(100) from equilibrium segregation studies. *Surf. Sci.* **1975**, *47*, 645–649, DOI: 10.1016/0039-6028(75)90208-3.

(6) Lang, B.; Joyner, R. W.; Somorjai, G. A. Low energy electron diffraction studies of chemisorbed gases on stepped surfaces of platinum. *Surf. Sci.* **1972**, *30*, 454–474, DOI: 10.1016/0039-6028(72)90012-X.

(7) Smoluchowski, R. Anisotropy of the Electronic Work Function of Metals. *Phys. Rev.* **1941**, *60*, 661–674, DOI: 10.1103/PhysRev.60.661.

(8) Pang, S. H.; Medlin, J. W. Adsorption and Reaction of Furfural and Furfuryl Alcohol on

Pd(111): Unique Reaction Pathways for Multifunctional Reagents. *ACS Catal.* **2011**, *1*, 1272–1283, DOI: 10.1021/cs200226h.

(9) Zhou, K.; Chen, J.; Cheng, Y.; Chen, Z.; Kang, S.; Cai, Z.; Xu, Y.; Wei, J. Enhanced Catalytic Transfer Hydrogenation of Biomass-Based Furfural into 2-Methylfuran over Multifunctional Cu–Re Bimetallic Catalysts. *ACS Sustainable Chem. Eng.* **2020**, *8*, 16624–16636, DOI: 10.1021/acssuschemeng.0c06026.

(10) High Resolution XPS of Organic Polymers: The Scienta ESCA300 Database (Beamson, G.; Briggs, D.). 1993; [Online; accessed 9. Jan. 2025].

(11) Beamson, G.; Clark, D. T.; Hayes, N. W.; Law, D. S.-L. Effect of Crystallinity on the XPS Spectrum of Poly(Ethylene Terephthalate). *Surf. Sci. Spectra* **1994**, *3*, 357–365, DOI: 10.1116/1.1247788.

(12) Tanuma, S.; Powell, C. J.; Penn, D. R. Calculations of electron inelastic mean free paths. V. Data for 14 organic compounds over the 50–2000 eV range. *Surf. Interface Anal.* **1994**, *21*, 165–176, DOI: 10.1002/sia.740210302.

(13) Tanuma, S.; Powell, C. J.; Penn, D. R. Calculation of electron inelastic mean free paths (IMFPs) VII. Reliability of the TPP-2M IMFP predictive equation. *Surf. Interface Anal.* **2003**, *35*, 268–275, DOI: 10.1002/sia.1526.

(14) Akhter, S.; White, J. M. Stabilization of C2Dx fragments by CO on Ni(100). *Surf. Sci.* **1987**, *180*, 19–46, DOI: 10.1016/0039-6028(87)90035-5.

(15) Benndorf, C.; Meyer, L. CO adsorption on stepped Ni(111) surfaces. *J. Vac. Sci. Technol., A* **1990**, *8*, 2677–2681, DOI: 10.1116/1.576693.

(16) MacIntosh, K. L.; Beaumont, S. K. Nickel-Catalysed Vapour-Phase Hydrogenation of Furfural, Insights into Reactivity and Deactivation. *Top. Catal.* **2020**, *63*, 1446–1462, DOI: 10.1007/s11244-020-01341-9.

(17) Lin, L.; Lai, M.; Li, H.; Tian, F.; Chen, Y.; Sun, J.; Lin, J.-M. Investigation of carbon deposition induced by pyrolytic decomposition of ethylene. *RSC Adv.* **2017**, *7*, 29639–29644, DOI: 10.1039/C7RA04282E.

(18) Lenk, T.; Rabet, S.; Sprick, M.; Raabe, G.; Schröder, U. Insight into the Interaction of Furfural with Metallic Surfaces in the Electrochemical Hydrogenation Process. *ChemPhysChem* **2023**, *24*, e202200614, DOI: 10.1002/cphc.202200614.

(19) Tsatsos, S.; Ladas, S.; Kyriakou, G. Electronic Properties and Reactivity of Furfural on a Model Pt(111) Catalytic Surface. *J. Phys. Chem. C* **2020**, *124*, 26268–26278, DOI: 10.1021/acs.jpcc.0c07709.

(20) Gordon, M. S.; Schmidt, M. W. *Theory and Applications of Computational Chemistry*; Elsevier: Walthm, MA, USA, 2005; pp 1167–1189, DOI: 10.1016/B978-044451719-7/50084-6.

(21) Schmidt, M. W.; Baldridge, K. K.; Boatz, J. A.; Elbert, S. T.; Gordon, M. S.; Jensen, J. H.; Koseki, S.; Matsunaga, N.; Nguyen, K. A.; Su, S.; Windus, T. L.; Dupuis, M.; Montgomery, J. A. General atomic and molecular electronic structure system. *J. Comput. Chem.* **1993**, *14*, 1347–1363, DOI: 10.1002/jcc.540141112.

(22) Kang, H.; Kasi, S. R.; Grizzi, O.; Rabalais, J. W. Interactions of low energy reactive ions with surfaces. II. Reactions of C+, O+, and CO+ ions with nickel oxide, carbide, and carbonyl. *J. Chem. Phys.* **1988**, *88*, 5894–5901, DOI: 10.1063/1.454522.

(23) Kuhlenbeck, H.; Neumann, M.; Freund, H.-J. Electronic and geometric structure of CO on Ni(110): Experiment and theory. *Surf. Sci.* **1986**, *173*, 194–214, DOI: 10.1016/0039-6028(86)90116-0.

(24) Gonze, X. et al. The Abinitproject: Impact, environment and recent developments. *Comput. Phys. Commun.* **2020**, *248*, 107042, DOI: 10.1016/j.cpc.2019.107042.

(25) Romero, A. H. et al. ABINIT: Overview and focus on selected capabilities. *J. Chem. Phys.* **2020**, *152*, 124102, DOI: 10.1063/1.5144261.

(26) Gonze, X. et al. Recent developments in the ABINIT software package. *Comput. Phys. Commun.* **2016**, *205*, 106–131, DOI: 10.1016/j.cpc.2016.04.003.

(27) Perdew, J. P.; Burke, K.; Ernzerhof, M. Generalized Gradient Approximation Made Simple. *Phys. Rev. Lett.* **1996**, *77*, 3865–3868, DOI: 10.1103/PhysRevLett.77.3865.

(28) Hamann, D. R. Optimized norm-conserving Vanderbilt pseudopotentials. *Phys. Rev. B* **2013**, *88*, 085117, DOI: 10.1103/PhysRevB.88.085117.

(29) Methfessel, M.; Paxton, A. T. High-precision sampling for Brillouin-zone integration in metals. *Phys. Rev. B* **1989**, *40*, 3616–3621, DOI: 10.1103/PhysRevB.40.3616.

(30) Lemmen, C.; Lengauer, T. Computational methods for the structural alignment of molecules. *J. Comput.-Aided Mol. Des.* **2000**, *14*, 215–232, DOI: 10.1023/a:1008194019144.

(31) Calle-Vallejo, F. The ABC of Generalized Coordination Numbers and Their Use as a Descriptor in Electrocatalysis. *Adv. Sci.* **2023**, *10*, 2207644, DOI: 10.1002/advs.202207644.
